# Supplementary figures and images for: Cellular morphodynamics as quantifiers for functional states of resident tissue macrophages in vivo
Source: PLoS Comput Biol. 2025 May 29;21(5):e1011859. doi: 10.1371/journal.pcbi.1011859 (PMC12193801; doi:10.1371/journal.pcbi.1011859)

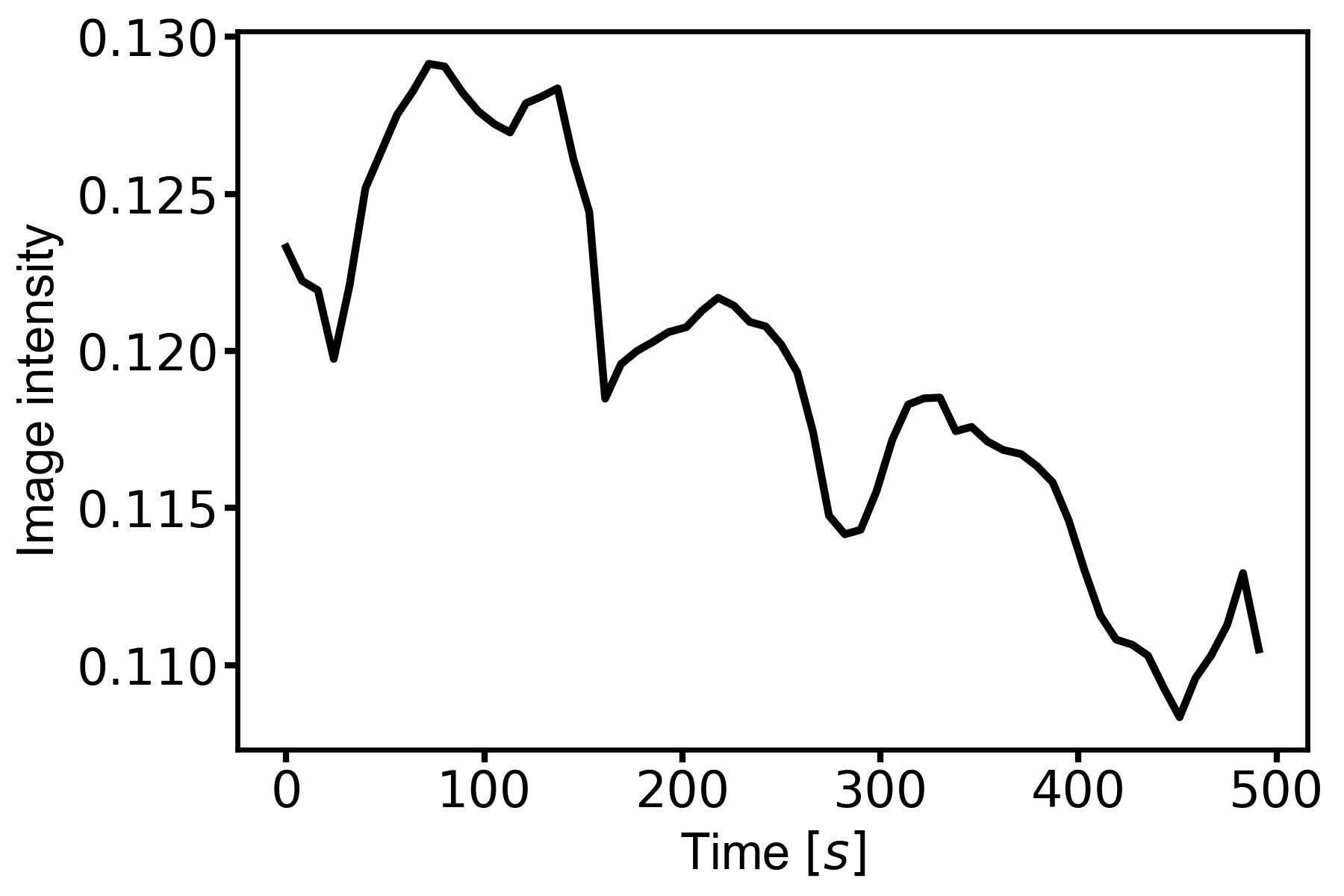

Supplement: S1 Fig — (PNG) [file pcbi.1011859.s001.png]

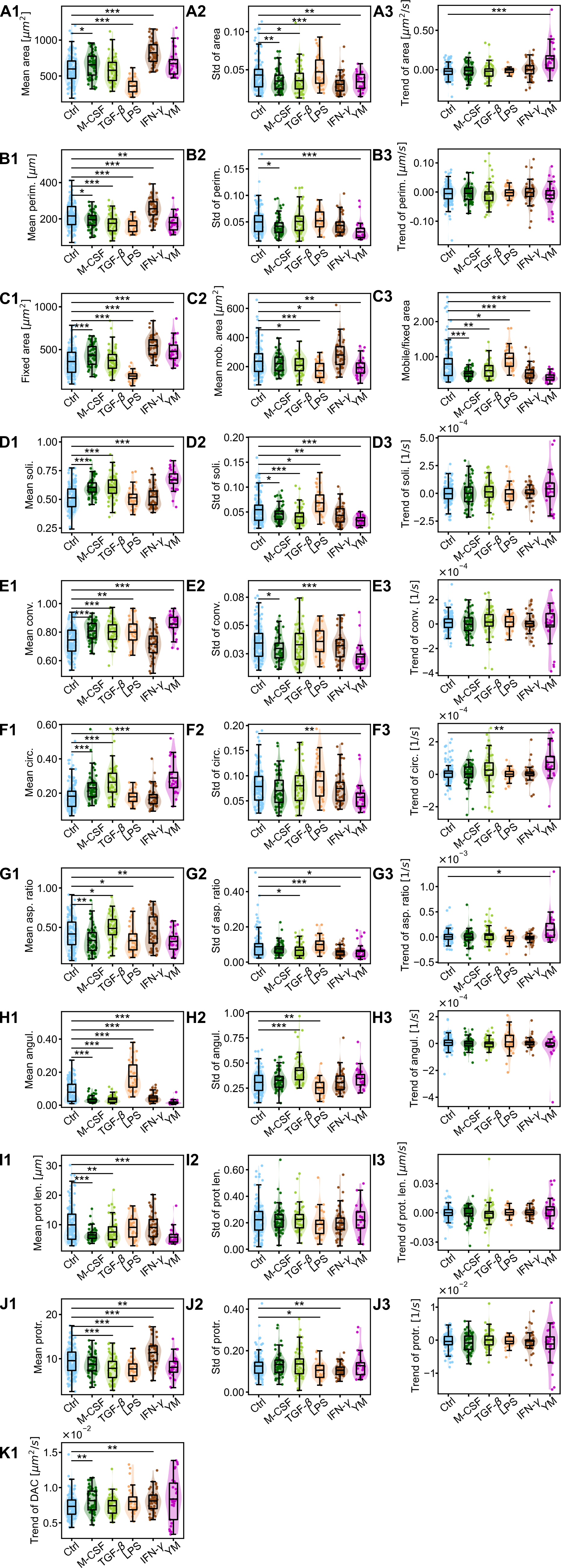

Supplement: S2 Fig — The boxplots show an extensive analysis of RTMs in vivo subjected to different chemical stimuli, as also described in Fig 4 - the different stimulants are: Unstimulated (Ctrl, N = 124), Macrophage Colony Stimulating Factor (M-CSF, N = 57), Transforming Growth Factor β (TGF-β, N = 53), Lipopolysaccharides (LPS, N = 32), Interferon γ (IFN-γ, N = 45), YM201636 (YM, N = 29). (A1) Mean of cell area. (A2) Standard deviation of cell area. (A3) Slope of a linear fit to cell area. (B1) Mean of cell perimeter. (B2) Standard deviation of cell perimeter. (B3) Slope of a linear fit to cell perimeter. (C1) Fixed cell area. (C2) Mobile cell area. (C3) Ratio of the mobile to the fixed cell area. (D1) Mean of solidity. (D2) Standard deviation of solidity. (D3) Slope of a linear fit to solidity. (E1) Mean of convexity. (E2) Standard deviation of convexity. (E3) Slope of a linear fit to convexity. (F1) Mean of circularity. (F2) Standard deviation of circularity. (F3) Slope of a linear fit to circularity. (G1) Mean of aspect ratio. (G2) Standard deviation of aspect ratio. (G3) Slope of a linear fit to aspect ratio. (H1) Mean of angularity. (H2) Standard deviation of angularity. (H3) Slope of a linear fit to angularity. (I1) Mean of the maximal protrusion length. (I2) Standard deviation of the maximal protrusion length. (I3) Slope of a linear fit to the maximal protrusion length. (J1) Mean of protrusiveness. (J2) Standard deviation of protrusiveness. (J3) Slope of a linear fit to protrusiveness. (K1) Trend of the dynamic area changes. The mean of the population is marked, tests for statistical significance were performed using a two-sided permutation Welch’s t-test. Significance is abbreviated as *p≤0.05, **p≤0.01, ***p≤0.001. (TIF) [file pcbi.1011859.s002.tif]

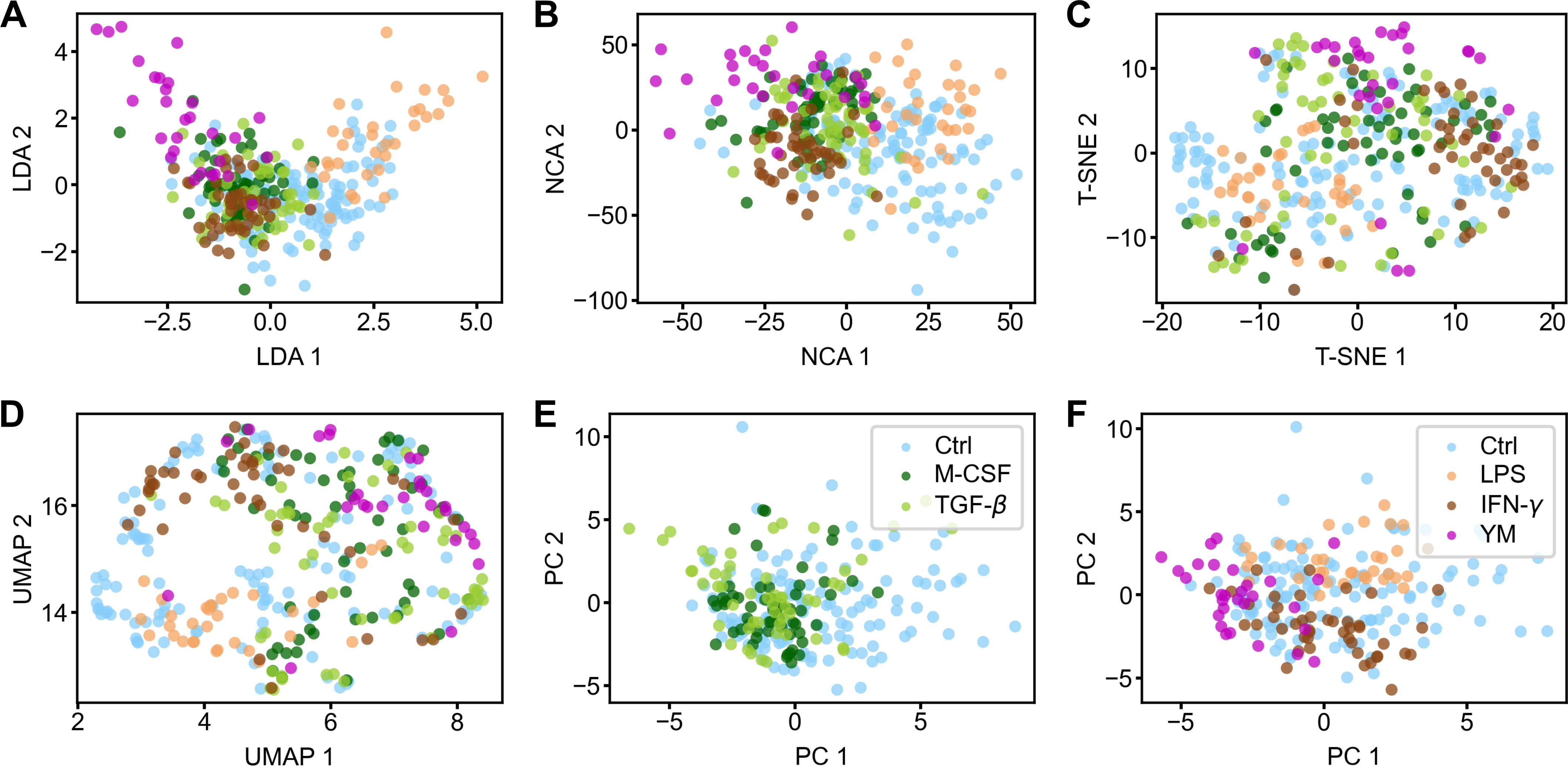

Supplement: S3 Fig — (A)–(F): Colors are blue for Control, dark green for M-CSF, yellowgreen for TGF-β, orange for LPS, brown for INF-γ and magenta for YM201636. (A)–(D): the dimensionality reduction methods were applied on all six cell populations. (A) Linear Discriminant Analysis. (B) Neighborhood Components Analysis. (C) T-distributed Stochastic Neighbor Embedding. (D) Uniform Manifold Approximation and Projection. (E) Principal Components Analysis using only Ctrl, M-CSF and TGF-β cell populations. (F) Principal Components Analysis, using only Ctrl, LPS, IFN-γ and YM201636 cell populations. (TIF) [file pcbi.1011859.s003.tif]

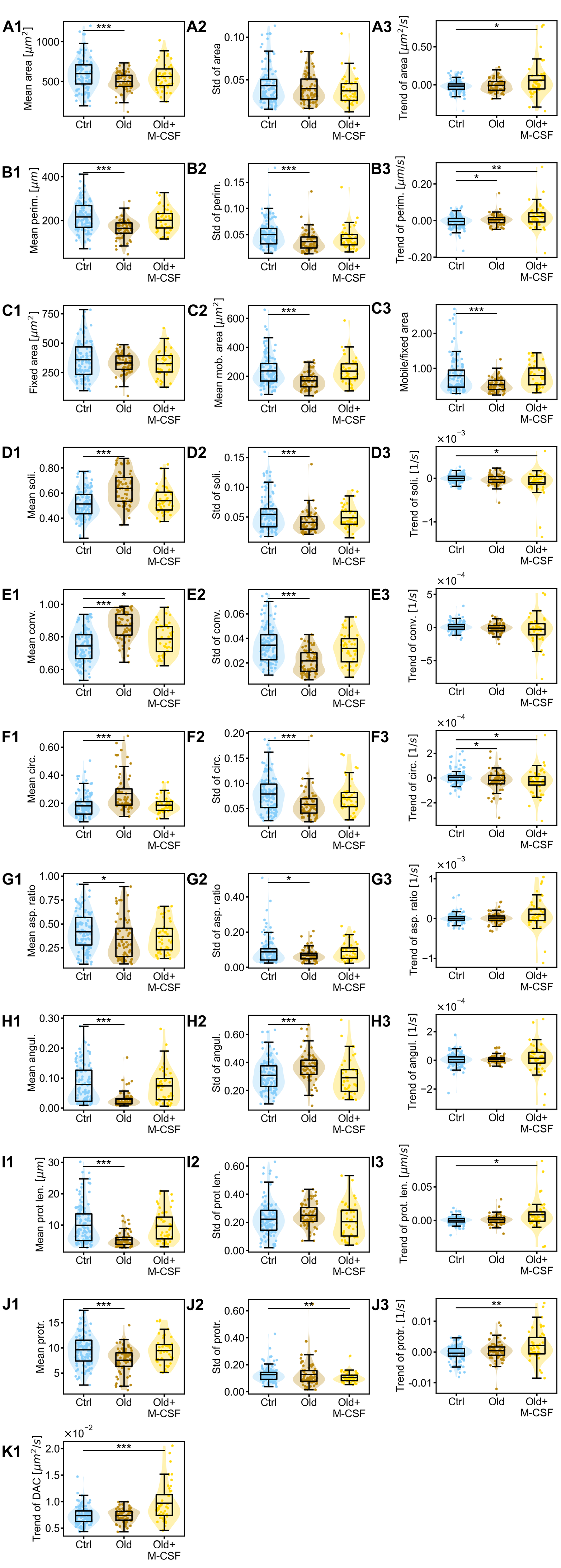

Supplement: S4 Fig — The RTMs of young mice (2–3 weeks) serve as control population (Ctrl, N = 124), while the RTMs of old mice (1 year) are compared either unstimulated (Old, N = 70), or stimulated with M-CSF (Old+M-CSF, N = 44). (A1) Mean of cell area. (A2) Standard deviation of cell area. (A3) Slope of a linear fit to cell area. (B1) Mean of cell perimeter. (B2) Standard deviation of cell perimeter. (B3) Slope of a linear fit to cell perimeter. (C1) Fixed cell area. (C2) Mobile cell area. (C3) Ratio of the mobile to the fixed cell area. (D1) Mean of solidity. (D2) Standard deviation of solidity. (D3) Slope of a linear fit to solidity. (E1) Mean of convexity. (E2) Standard deviation of convexity. (E3) Slope of a linear fit to convexity. (F1) Mean of circularity. (F2) Standard deviation of circularity. (F3) Slope of a linear fit to circularity. (G1) Mean of aspect ratio. (G2) Standard deviation of aspect ratio. (G3) Slope of a linear fit to aspect ratio. (H1) Mean of angularity. (H2) Standard deviation of angularity. (H3) Slope of a linear fit to angularity. (I1) Mean of the maximal protrusion length. (I2) Standard deviation of the maximal protrusion length. (I3) Slope of a linear fit to the maximal protrusion length. (J1) Mean of protrusiveness. (J2) Standard deviation of protrusiveness. (J3) Slope of a linear fit to protrusiveness. (K1) Trend of the dynamic area changes. The mean of the population is marked, tests for statistical significance were performed using a two-sided permutation Welch’s t-test. Significance is abbreviated as *p≤0.05, **p≤0.01, ***p≤0.001. (TIF) [file pcbi.1011859.s004.tif]

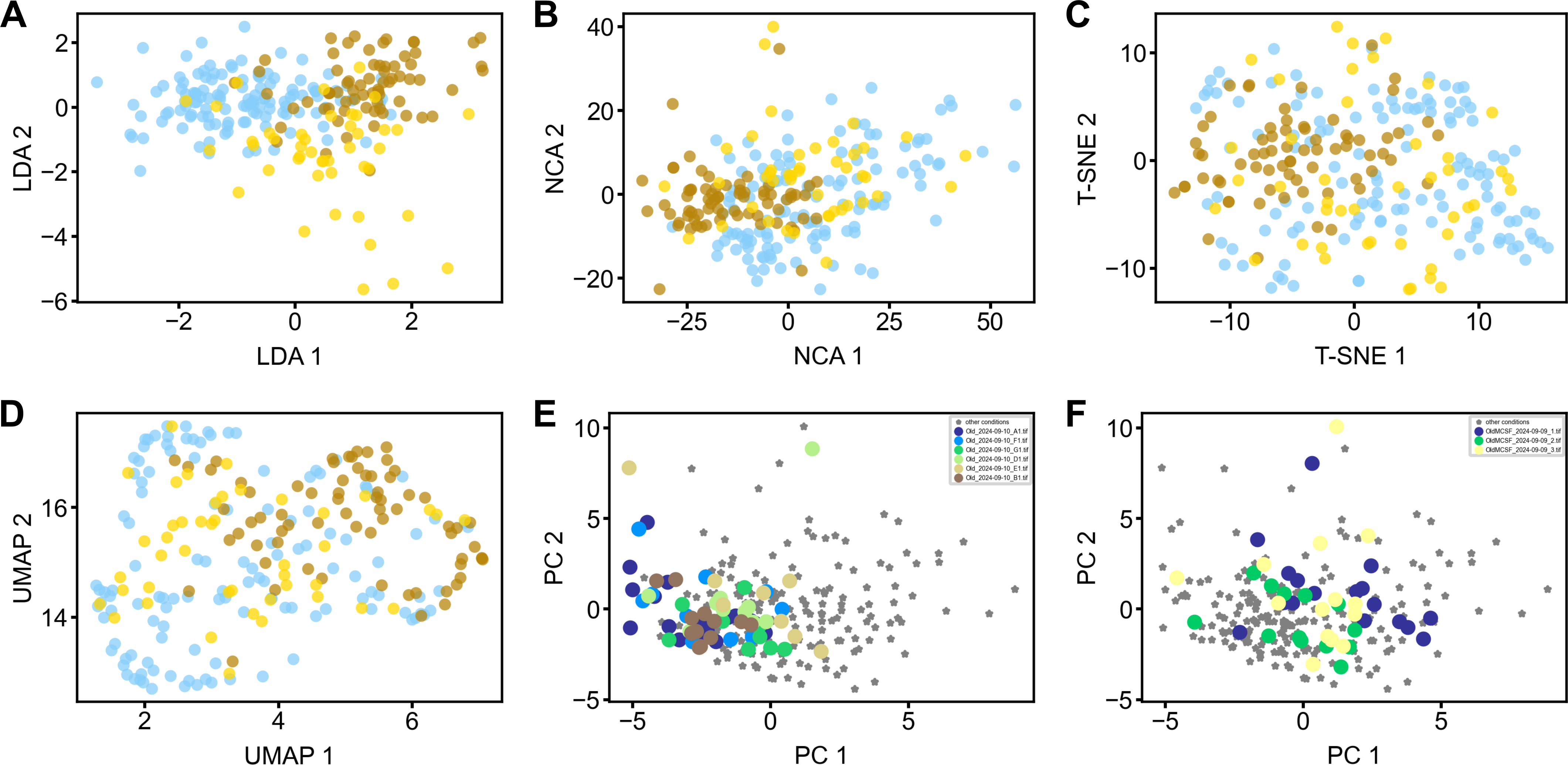

Supplement: S5 Fig — (A)–(D): Colors are blue for Control, dark gold for old RTMs and yellow for old RTMs stimulated with M-CSF. (A) Linear Discriminant Analysis. (B) Neighborhood Components Analysis. (C) T-distributed Stochastic Neighbor Embedding. (D) Uniform Manifold Approximation and Projection. (E) Principal Components Analysis, where the cells from the old population are marked according to their experiment - Ctrl and Old+M-CSF cells are marked as stars. (F) Principal Components Analysis, where the cells from the Old+M-CSF population are marked according to their experiment - Ctrl and Old cells are marked as stars. (TIF) [file pcbi.1011859.s005.tif]

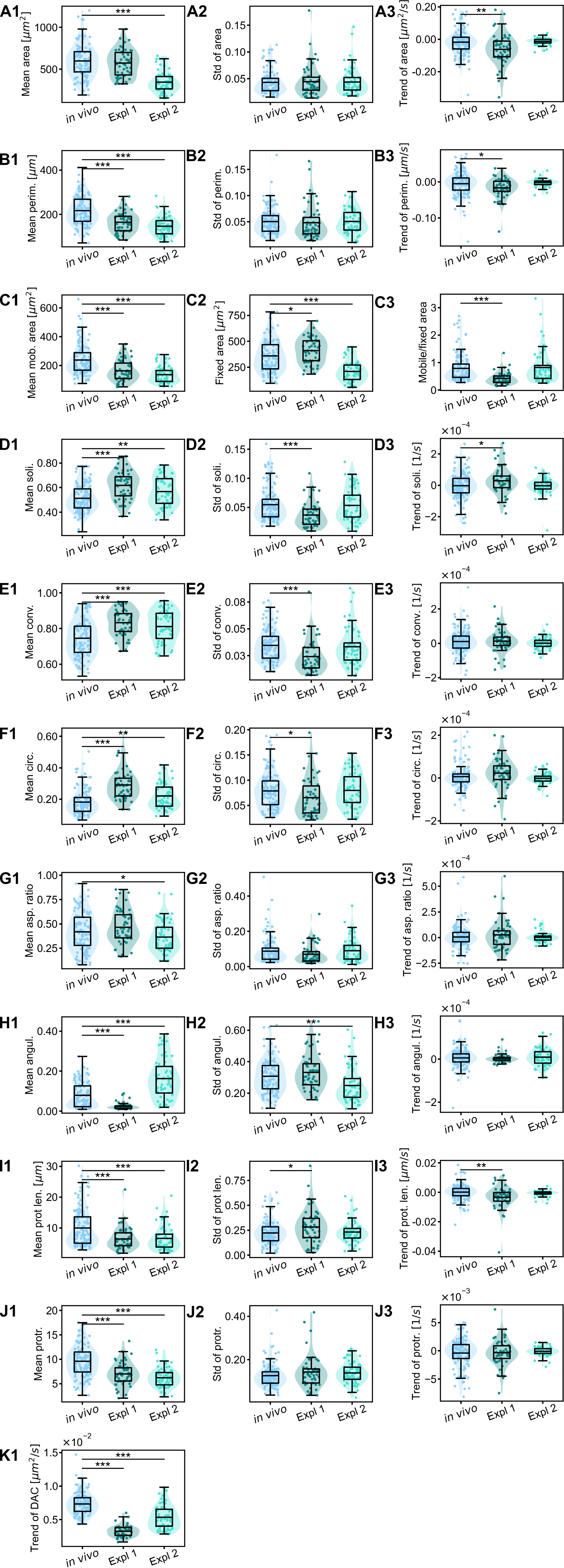

Supplement: S6 Fig — The cells imaged in vivo serve as control, explanted cells were imaged using two different media (Expl 1 and Expl 2). (A1) Mean of cell area. (A2) Standard deviation of cell area. (A3) Slope of a linear fit to cell area. (B1) Mean of cell perimeter. (B2) Standard deviation of cell perimeter. (B3) Slope of a linear fit to cell perimeter. (C1) Fixed cell area. (C2) Mobile cell area. (C3) Ratio of the mobile to the fixed cell area. (D1) Mean of solidity. (D2) Standard deviation of solidity. (D3) Slope of a linear fit to solidity. (E1) Mean of convexity. (E2) Standard deviation of convexity. (E3) Slope of a linear fit to convexity. (F1) Mean of circularity. (F2) Standard deviation of circularity. (F3) Slope of a linear fit to circularity. (G1) Mean of aspect ratio. (G2) Standard deviation of aspect ratio. (G3) Slope of a linear fit to aspect ratio. (H1) Mean of angularity. (H2) Standard deviation of angularity. (H3) Slope of a linear fit to angularity. (I1) Mean of the maximal protrusion length. (I2) Standard deviation of the maximal protrusion length. (I3) Slope of a linear fit to the maximal protrusion length. (J1) Mean of protrusiveness. (J2) Standard deviation of protrusiveness. (J3) Slope of a linear fit to protrusiveness. (K1) Trend of the dynamic area changes. The mean of the population is marked, tests for statistical significance were performed using a two-sided permutation Welch’s t-test. Significance is abbreviated as *p≤0.05, **p≤0.01, ***p≤0.001. (TIF) [file pcbi.1011859.s006.tif]

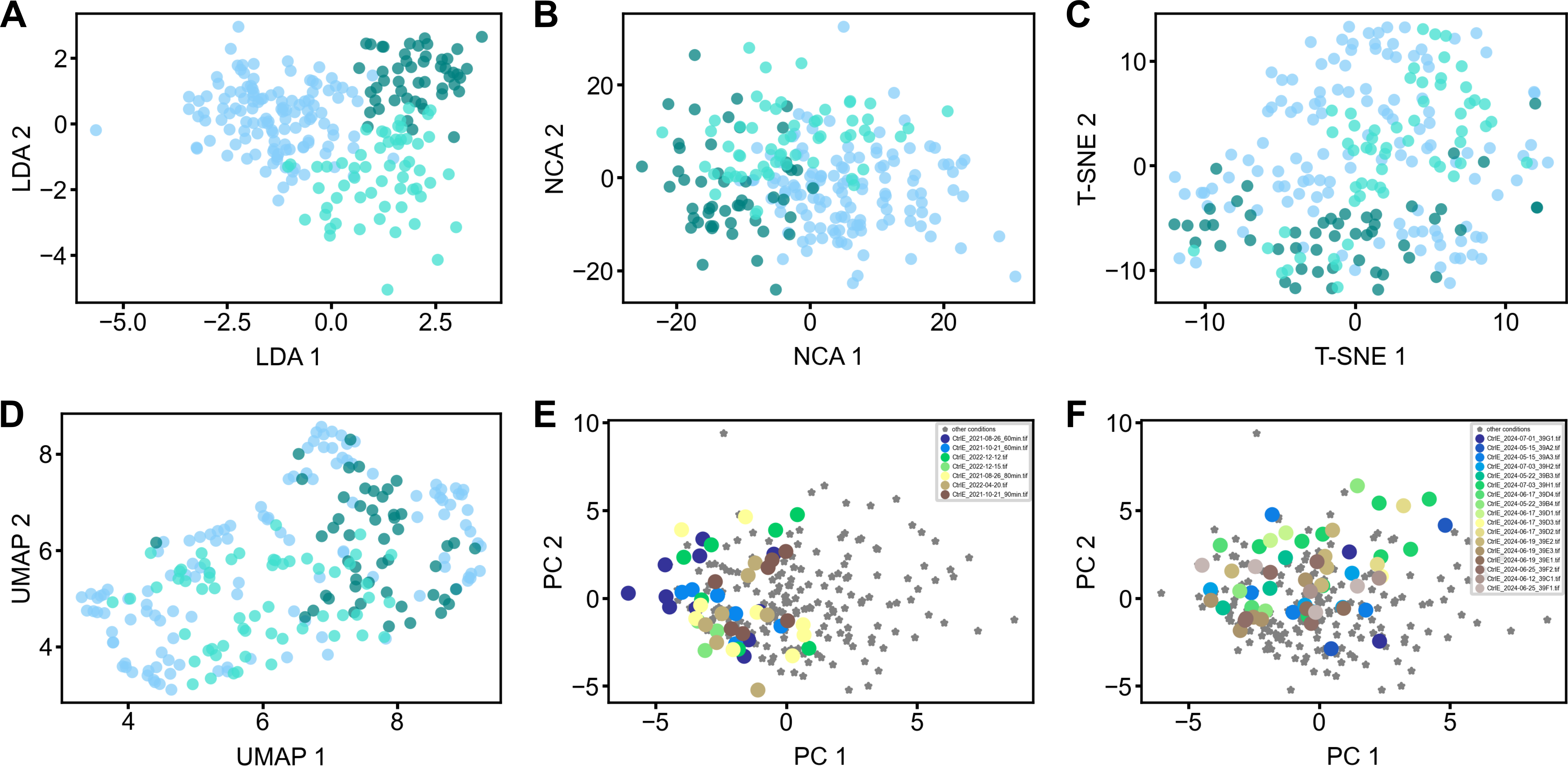

Supplement: S7 Fig — (A)–(D): Colors are blue for in vivo control, teal/dark greenish for Explant 1, turquoise/light greenish for Explant 2. (A) Linear Discriminant Analysis. (B) Neighborhood Components Analysis. (C) T-distributed Stochastic Neighbor Embedding. (D) Uniform Manifold Approximation and Projection. (E) Principal Components Analysis, where the in vivo cell population and the Explant 2 cell populations are marked as stars whereas the Explant 1 cell population is colored according to their corresponding experiment. (F) Principal Components Analysis, where the in vivo cell population and the Explant 1 cell populations are marked as stars whereas the Explant 2 cell population is colored according to their corresponding experiment. (TIF) [file pcbi.1011859.s007.tif]
