# Supplementary material for: Cellular morphodynamics as quantifiers for functional states of resident tissue macrophages in vivo
Source: PLoS Comput Biol. 2025 May 29;21(5):e1011859. doi: 10.1371/journal.pcbi.1011859 (PMC12193801; doi:10.1371/journal.pcbi.1011859)
Supplement: S1 Text — (PDF) [file pcbi.1011859.s010.pdf]

Supplemental material to "Cellular morphodynamics as quantifiers for functional states of resident tissue macrophages *in vivo*"

Miriam Schnitzerlein, Eric Greto, Anja Wegner, Anna Möller, Oliver Aust, Oumaima Ben Brahim, Vasily Zaburdaev, Stefan Uderhardt

Interpretation of morphology quantifiers

In our manuscript, we use several cell size and shape quantifiers to assess morphodynamical changes of resident tissue macrophages (RTMs) under various conditions. The chosen quantifiers are not completely independent of each other, and one might wonder if it is necessary to measure all of them. However often different shapes which have the same value for some quantifiers can still be differentiated using others. The interplay of the quantifiers when considering varying cell shapes is examined in more detail below.

Cell area and perimeter

The cell area can increase/ decrease depending on whether the cells ingest/ secrete particles or extracellular fluid. In principle, the (two-dimensional) cell area can also change if the (three-dimensional) cell volume is reorganized such that the projection of the volume changes significantly. This option is rarely relevant for the case of resident tissue macrophages in the peritoneal serosa as the tissue geometry does not allow the cells to grow significantly in the z-direction. Unphysiological ways to change the cell area include adding holes in the cell body (which reduces the cell area) or if the segmentation unites a superimposing, migratory cell to the original cell area - those events are excluded from happening due to a quality control of the labeled cells of our experiments.

The cell perimeter can of course change with the cell area, as a larger cell might also have a larger cell boundary. However, in our case, the cell boundary mostly changes due to cell shape changes, which will be considered in more detail in the following

Solidity

The solidity of an object is defined as

$$\text{Solidity} = \frac{\text{object area}}{\text{convex area}}$$

and can take values between 0 and 1. A solidity of 1 thereby signifies an object which is perfectly convex, meaning that it has no inward or outward bulges. Examples for convex objects are circles, ellipses, squares, triangles or rectangles. When the solidity of an object goes to 0 it would mean that the area of the object goes to 0 while its convex area goes to infinity. Here, one can imagine a very thin spider web spanning over a large space. Such extreme cases rarely describe physiological cell morphologies of RTMs.

Changes in solidity of a cell will be differentiated here in two cases:

- **Roughly constant cell area.** When the cell area stays constant, the solidity can only change if the cell rearranges internally (Fig 1 B and D).

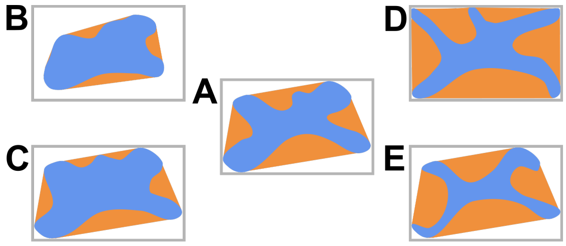

Figure 1: Sketches showing an exemplary cell (A) with different solidities (B-E). The cell area is colored blue, while its convex area is colored orange. The added gray frames all have the same size to simplify size comparison of the sketches.

  - By retracting its protrusions and thus 'thickening' the cell body, its convex area decreases and thus the cells solidity increases, as shown in Fig 1 B. Furthermore, the cell perimeter, convexity, protrusiveness and maximum length of protrusions will be decreased, while the circularity will be increased.
  - By extending and simultaneously thinning its protrusions, the cell spreads over a larger space and the solidity decreases, see Fig 1 D. A morphology change like this will be accompanied by a decrease in circularity and in increased perimeter, protrusiveness and maximum protrusion length.

- **Changing cell area.** If the cell area is changing, but the convex area isn't, the following two scenarios can happen:
  - If the cell area is increasing, thus progressively filling the convex outline of the cell, the cell solidity will be increasing as exemplary shown in Fig 1 C. Such a morphology change would - similarly to the case in Fig 1 B - result in a decreased convexity and protrusiveness, while the circularity would again be increased.
  - If the cell area is decreasing in a way that the cell is thinning its protrusions, the cell solidity will be decreasing (see Fig 1 E).

Of course, both the cell area and the convex area can change simultaneously - if they change at a similar rate with the same sign, the solidity should stay roughly constant. Conversely, if either cell area or convex area increases while the other decreases, the solidity changes accordingly, and the resulting morphological change can be expected to be a more extreme case of the sketches presented in Fig 1 B-E.

Convexity

The convexity of an object is defined very similarly to the solidity, but considering the cell perimeter, namely

$$\text{Convexity} = \frac{\text{convex perimeter}}{\text{object perimeter}}$$

and can take values between 0 and 1. A convexity of 1 thereby signifies again an object which is perfectly convex, meaning that it has no inward or outward bulges. When the convexity of an object goes to 0 it would mean that the convex perimeter of the object is very small while its perimeter goes to infinity. An example might be a snowflake-like object, which can have a very intricate shape with fern-like stellar dendrites, while its convex outline is comparatively small and circular.

- **Increasing convexity.** The convexity can increase either by

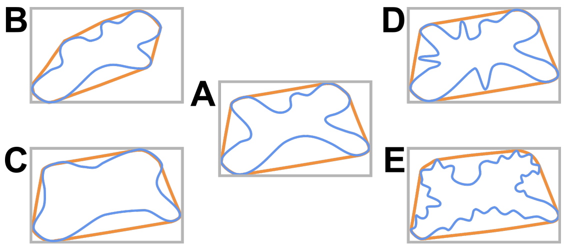

Figure 2: Sketches showing an exemplary cell (A) with different convexities (B-E). The cell perimeter is colored blue, while its convex perimeter is colored orange. The added gray frames all have the same size to simplify size comparison of the sketches.

  - Decreasing the convex perimeter as in Fig 2 B via retracting some of the cell protrusions. This will result in a potential increase of the cell solidity and circularity and a decrease of the area, protrusiveness and maximal protrusion length, while the other parameters do not necessarily need to change in this example.
  - By decreasing the cell perimeter via growing into the inward bulges, the convexity will also increase as shown in Fig 2 C. Similarly to Fig 2 B, an increase of cell solidity and circularity and a decrease of the protrusiveness is expected. However, opposite to Fig 2 B, the cell here would have an increased cell area and the maximal protrusion length might stay constant or be even increased.

- **Decreasing convexity.** Essentially, the cell boundary needs to become more complex, i.e. the cell perimeter needs to grow while the convex perimeter stays constant (or even shrinks).
  - The cell perimeter can increase as the cell grows out new protrusions as visualized in Fig 2 D. Then also the cell area, solidity and protrusiveness should be increased, while the circularity should be decreased.
  - The convexity will also decrease if the roughness of the cell boundary, as sketched in Fig 2 E, will increase. In this case, the cell area and solidity are not necessarily changed, but the circularity will be decreased while the angularity will be increased.

Circularity

The circularity of an object is defines as

$$\text{Circularity} = \frac{4\,\pi \cdot \text{object area}}{(\text{object perimeter})^2}$$

which can again take values between 0 and 1. A circularity of 1 corresponds to a perfect circle, while for an object with circularity going to 0 its area decreases while its perimeter increases.

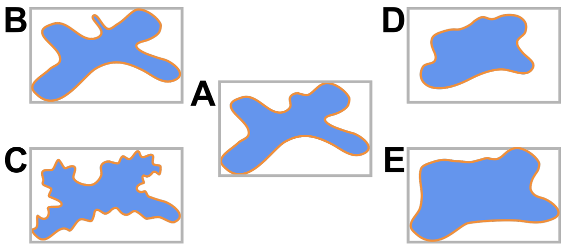

Figure 3: Sketches showing an exemplary cell (A) with different circularities (B-E). The cell body (area) is colored blue, while its boundary (perimeter) is colored orange. The added gray frames all have the same size to simplify size comparison of the sketches.

Morphodynamic changes that lead to a change in circularity - starting from a cell shape as shown in Fig 3 A - can be categorized as follows:

- **Decreasing circularity.** For the circularity to decrease, either the cell area has to decrease (with constant perimeter) or the perimeter has to increase (with constant area), or of course both at the same time. For the specific case of macrophages, one can think of two main scenarios - though combinations can also exist:
  - The cell perimeter increases due to the growth of new protrusions as visualized in Fig 3 B, thus increasing its perimeter. Such a change should be accompanied by a change in protrusiveness.
  - The membrane of the cell becomes rougher or more wrinkly as exemplified in Fig 3 C, thus increasing the cell perimeter while the cell area can stay constant. Here, an increase of the angularity and a decrease of convexity would be expected.
- **Increasing circularity.** For the circularity to increase, the cell area has to increase or the cell perimeter has to decrease (or both at the same time).
  - The cell can retract its protrusions and organize the available area closer to its cell body (see e.g. Fig 3 D), thus decreasing the cell perimeter while (potentially) keeping the area constant. Such a morphology change would be accompanied by an increase of solidity and convexity, as well as a decrease of the cell perimeter and protrusiveness.
  - Conversely, the cell could grow to fill the grooves by increasing its area, which would decrease its perimeter (see Fig 3 E). Similar to Fig 3 D, such a cell change would be accompanied by an increase of solidity and convexity, as well as a decrease of the cell perimeter and protrusiveness, though here an additional increase of cell area is expected.

Aspect ratio

For the aspect ratio, an ellipse with the same first moments is fitted to the object of interest, and the major (i.e. longer) and minor (i.e. smaller) axis of that ellipse are compared as

$$\text{Aspect ratio} = \frac{\text{minor axis}}{\text{major axis}}.$$

The aspect ratio can take values between 0 and 1 - here 1 signifies an object where both axes have the same length, like a circle or a square. An aspect ratio of 0 would correspond to an object where the minor axis is negligible compared to the length of the major axis, like a very long, but thin line.

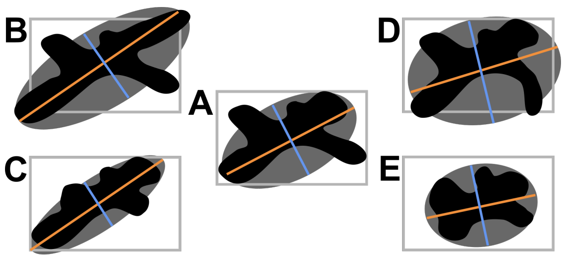

Figure 4: Sketches showing an exemplary cell (A) with different aspect ratios (B-E). The cell body (area) is colored black, while a gray ellipse with the same moments as the cell is added in the background. The major axis is marked in orange and the minor axis is marked in blue. The added gray frames all have the same size to simplify size comparison of the sketches.

The aspect ratio changes when either the major or the minor axis of an object changes.

- **Increasing aspect ratio.** If the aspect ratio increases, the lengths of the two axes become more comparable.

- The minor axis can grow in length which happens if the cell grows along that axis. The change of aspect ratio is independent of whether the cell only grows single protrusions (as shown in Fig 4 D, potentially increasing the protrusiveness and decreasing the circularity), or if it grows similar as in Fig 3 E, thus solidifying and decreasing its protrusiveness. Importantly, a larger aspect ratio does not necessarily signify a rounder or more solid cell.
- Conversely, the major axis could shrink to increase the aspect ratio - typically by retracting its protrusions as shown in Fig 4 E.

- **Decreasing aspect ratio.** If the aspect ratio decreases, the difference of lengths of the two axes increase.
  - The major axis can grow in length if the cell extends its protrusions as shown in Fig 4 B.
  - If the minor axis shrinks in length due to a retraction of protrusions, the aspect ratio will also decrease. Such a change will most likely be accompanied by an increase in solidity and decrease of protrusiveness. Of course, the two effects can be combined (i.e. growing protrusions along one axis while retracting protrusions along the other) to achieve a larger change of the aspect ratio.

Angularity

For the angularity, the object boundary is smoothed using an erosion-dilation operation, which is also known as opening in morphological image analysis. Here (small) irregularities of the object boundary - depending on the amount of erosions used respectively the size of the structuring element used in the erosion - are being removed, thus one achieves a smoother object boundary. Then, this smoothed object area is subtracted from the original object area and subsequently normalized by the original object area. Thus the angularity measures the ratio of wrinkly object area along the object outline compared to the total object area:

$$\text{Angularity} = \frac{\text{object area} - \text{smoothed object area}}{\text{object area}}$$

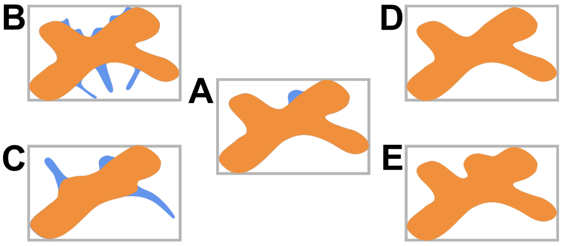

Figure 5: Sketches showing an exemplary cell (A) with different angularities (B-E). The original cell body (area) is colored blue, while a smoothed version of the cell body is colored in orange. The added gray frames all have the same size to simplify size comparison of the sketches.

- **Increasing angularity.** If the angularity increases, the cell boundary becomes more irregular, which can be achieved in multiple ways:
  - Many small irregularities are added to the cell boundary (see Fig 5 B at the top of the cell). Such a morphology change would be accompanied by a decrease of circularity (see Fig 3 C) and convexity (see Fig 2 E).

- Thin, but longer protrusions are growing from the cell body which are still thin enough to be removed by the erosion operation (see Fig 5 B at the bottom of the cell). In that case, a decrease of circularity and convexity might also be measured, as well as a potential decrease of solidity.
- Protrusions, which were previously large enough to be unaffected by the erosion-dilation might become thinner, thus being removed during the erosion (see Fig 5 C). Importantly, in such a case the other cell shape parameters would be largely unaffected and can stay the same, while the angularity can change quite dramatically.

- **Decreasing angularity.** For the angularity to decrease, its cell boundary needs to be more smooth. That can be achieved by either
  - removing or shrinking smaller irregularities on the cell boundary, as shown in Fig 5 D or
  - growing such irregularities such that they are too large to be considered irregular and are thus not removed in the erosion-dilation process (see Fig 5 D).

Maximum protrusion length

For the maximum protrusion length, the fixed area of a cell first has to be determined. Then, the smallest distance from every boundary point to the fixed area is calculated - the maximum of those distances then signifies the length of the longest protrusion.

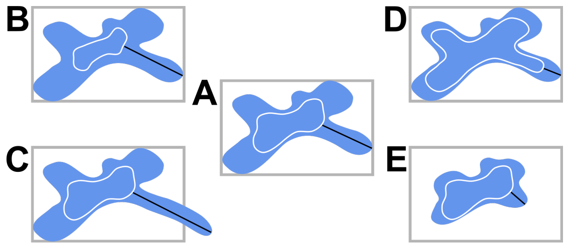

Figure 6: Sketches showing an exemplary cell (A) with different maximal protrusion lengths (B-E). The cell body is colored blue, while the outline of the fixed area is colored white and the length of the longest protrusion is marked in black. The added gray frames all have the same size to simplify size comparison of the sketches.

The length of the maximum protrusion is thus determined by the size and shape of the fixed area as well as the size and shape of the cell boundary.

- **Increasing maximum protrusion length.** The protrusion length can increase due to
  - a decrease of the fixed cell area (see Fig 6 B), which automatically increases the distance from cell boundary to fixed area. All other cell shape quantifiers would be unchanged in such a case, except for a change of the fixed area.
  - an outgrowth of the protrusion (see Fig 6 C). In that case, one could also measure a decrease of the solidity and circularity and an increase of protrusiveness.

- **Decreasing maximum protrusion length.** Similarly to the increasing protrusion length, a decrease can be due to
  - an increase of the fixed cell area (see Fig 6 D), which would again not change any of the other cell shape parameters, except for the fixed area.

– a retraction of the previous longest protrusion - or potentially all protrusions -, resulting in a global decrease of the maximal protrusion length (see Fig 6 E). That would most likely be accompanied by an increase in solidity, circularity and convexity as well as a decrease of protrusiveness.

Importantly, the cell shape could also change rather dramatically due to a 'reorganization' of the cell protrusions while the maximal protrusion length stays the same.

Protrusiveness

For measuring the protrusiveness, a Sholl analysis is used, where concentric circles (so called Sholl circles) are placed on the cell and the amount of intercepts of the cell body with the circles are summed up. Importantly, if a circles lies completely inside of the cell body, the 'intercept' will not be counted - else it would be impossible to differentiate between a very large, but circular cell and a small cell with many protrusions.

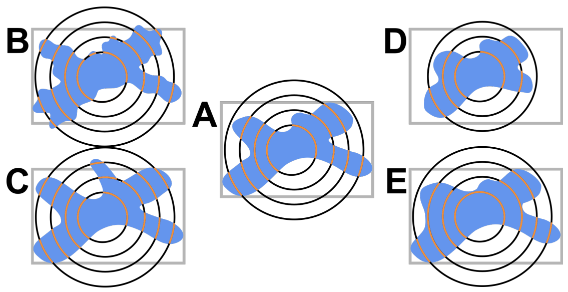

Figure 7: Sketches showing an exemplary cell (A) with different protrusivenesses (B-E). The cell body is colored blue, while concentric Sholl circles are colored in dark gray. The intercept of Sholl circles with the cell body is colored in orange. The added gray frames all have the same size to simplify size comparison of the sketches. As an example, the protusiveness of the cell in (A) is 11.

- **Increasing protrusiveness.** To increase the protrusiveness of a cell, the cell body has to intercept the Sholl circles more often.
  - One possibility if to increase boundary irregularities (see Fig 7 B) - if those irregularities are close to the Sholl circles, the protrusiveness will increase, as will the angularity and perimeter whereas the convexity should decrease.
  - If the cell grows more and longer protrusions, its protrusiveness will also increase as visualized in Fig 7 C - furthermore the cells solidity and circularity might be decreased.
- **Decreasing protrusiveness.** To decrease the protrusiveness, the cell either needs to intercept the Sholl circles less often, or to encircle the Sholl circle completely.
  - In Fig 7 D, the cell retracts its protrusions (partially), thus decreasing its protrusiveness. Such a morphology change would also be seen in an increased solidity, convexity and circularity, as well as a potentially decreased cell area and maximal protrusion length.
  - In Fig 7 E, the cell grows larger and thus reduces two previously separate intercepts to only one intercept. Here an area increase would be expected, additionally to an increase of solidity, convexity and circularity similar to Fig 7 D.

Interpretation of cell dynamics quantifiers

The cell area can be divided into a mobile and a fixed portion. While a larger mobile area should correspond to a more active cell, this parameter is highly dependent on the overall cell area, i.e.

a larger cell will have a larger mobile area. Therefore, the ratio of the mobile to fixed area is calculated.

The standard deviation measures the range of the cell morphology quantifiers - thus if the standard deviation of a feature is higher, the underlying features may fluctuate with a higher amplitude. Unfortunately, the standard deviation cannot inform about the speed at which the morphological changes occur, i.e. in the case of macrophages whether the cells are sampling faster or slower due to certain chemical stimuli.

The dynamic area change (DAC) gives more insight into the speed of sampling. To calculate it, the cell bodies at two time points are overlayed with each other and the area which is not shared between the two cells is summed up. Then, the trend of (i.e. slope of the linear fit to) the cumulative sum of the DAC is calculated which tells us how fast the cell can change its area between two time points. Furthermore we can see how much of the surrounding tissue the cell can sample up to a certain point in time. As the DAC is clearly highly correlated to the overall cell size - as a larger cell typically has a larger mobile area with which it can sample - the DAC is corrected by dividing it by the cell area. It is a parameter that should be completely independent from all other morphological parameters or cell shapes introduced in this manuscript.
