# Supplementary material for: Cellular morphodynamics as quantifiers for functional states of resident tissue macrophages in vivo
Source: PLoS Comput Biol. 2025 May 29;21(5):e1011859. doi: 10.1371/journal.pcbi.1011859 (PMC12193801; doi:10.1371/journal.pcbi.1011859)
Supplement: Attachment — Submitted filename: Schnitzerlein2024_PCBreview.pdf [file pcbi.1011859.s011.pdf]

This paper explores interesting questions about how the morphology of resident tissue macrophages relates to their functional state. The authors introduce a novel experimental and computational pipeline to extract morphological quantifiers from imaging data which is very interesting, and use this to calculate statistics relating to macrophages activated using different stimuli. This study would be of interest to readers of Plos Computational Biology.

I have some reservations about the scope of the morphological descriptors used here, which convert the rich time series data extracted by the authors into a small number of simple quantitative values. Figures 3 and 4 suggest to me that the statistics chosen do not capture macrophage morphology in sufficient detail to adequately distinguish between different shapes. In particular, I think it is important for the authors to clarify why they selected the subset of statistics that they have used, as I think it is likely that the descriptors could be improved substantially by either 1) including more complex descriptors of shape or 2) fully exploiting the time series data rather than simplifying it into simple linear trend.

<u>Main comments</u>

1. How correlated are the summary statistics chosen for this data? E.g. the curves in Fig 2D look highly correlated, how much value is added by measuring all these metrics?
2. Why were these 4 shape metrics chosen, and not other possible choices? For instance, several others are mentioned in the text, and there are a huge number of shape quantifiers available in the wider mathematical literature that are not discussed (e.g., fractal dimension as a measure of boundary complexity). Given that the metrics chosen here don't seem to distinguish well between different macrophage subtypes (see PCAs in Fig 3 and Fig 4 and comments 6/7 below), it seems strange not to include a wider range of quantifiers.
3. The move from time series of statistics to aggregate statistics is not well clarified here – for instance, in Fig 3 the mean of the perimeter is used, but the Std of solidity and circularity. Why were these metrics chosen? How well do they capture the variation in the time series? Why are linear trends of the time series taken when they are seen in Fig 2 to be cyclical?
4. Figure 3 is used to support a wide range of claims on pages 6-9, but much relevant information is instead in the SI (Fig S2) and it is not clear that the interpretations given here are the only way of interpreting these statistics. Clarifying the interpretations of the statistics used in this paper would be extremely helpful to demonstrating that these boxplots (most of which show no statistical significance) support the authors' interpretations regarding macrophage morphology. Otherwise we are left to rely on statements like "it was visually apparent that x" which are then supported with statistics that may

support this interpretation, or may support some other interpretation. See, for example, lines 238-241. Increased roundness is a possible interpretation of the changing perimeter etc mentioned here, but there is no discussion in the text as to whether another change in morphology could also give rise to these changes in statistics.

   a. See also page 7: “We have shown that our set of parameters were indeed capable of differentiating between RTMs perturbed by different chemical stimuli - some of the features do so on the population-level while others are more useful in direct comparison at the cell-level” – I don’t understand how Fig 3 supports this?

5. S2 Fig – S4 Fig – some captions would be extremely helpful in clarifying what these images show
6. Fig 3 panels I and J – how much of the variance do PC1 and PC2 explain? Would using 3 principal components lead to better separation between the classes? Does clustering in the high dimensional space (rather than the reduced dimensional projection) allow the clusters to separate?
7. The PCA plots shown in Fig 3I and Fig 4F both seem to show no substantial difference between the classes. It seems likely to me that this is because the features chosen to define the high dimensional space do not adequately describe shape differences present between the macrophages compared (see comment 2 above). The lack of significant differences in Fig 3 B-G seems to support this. How important are the features the authors have selected here? Why were these features chosen to summarise the time series in Fig 2 described and not others?
8. The authors describe their features repeatedly as “human-interpretable”. It would be useful to have some comment on how these features can be interpreted, particularly when interpreting combinations of shape quantifiers. What information does each quantifier give? When do they give similar or different information, and how does this affect the interpretation? (See also comment 4 around the interpretation of Fig 3.)

<u>Minor/pedantic comments</u>

1. Fig 2C: Although explained in more detail in the main text, I found the different area curves confusing here. Could they be colour coded to relate them to the relevant outlined areas in Fig 2A? If the fixed area is constant, what is gained by plotting both the total area and mobile area – is the total area just the sum of the other two curves, one of which is constant?
2. Page 2: “Although cell shape analyses have been used to characterize macrophages under various conditions, most of the approaches used were either not designed to assess the dynamics behavior of peripheral RTMs over time or did not take into account the inhere characteristics of sessile RTMs *in*

vivo." This sentence seems to be a brief discussion of previous literature, which is not cited here. Which previous approaches are the authors referring to? Can citations to them be added here?

3. Fig 1 provides a clear demonstration that in these data the macrophages observed *in vivo* and *in vitro* display different motile vs sessile behavior. Is this representative of all such *in vivo* vs *in vitro* phenotypes? Have any RTMs *in vivo* been observed to be motile, and do RTMs placed *in vitro* display a motile or sessile phenotype? Some minor expansion on the limit of these experiments (or supporting references here or in the introduction if this behavior is well-known in the literature) would be helpful to clarify.
4. The analysis is conducted in 2D, but 3D data is available, with a brief justification given for working with maximum intensity projections (page 4). It would be useful if possible to see a brief quantitative justification for this (e.g., supplementary images showing the symmetry in the z-direction, or quantification showing that including morphology in the z-direction does not add predictive value), especially given that lack of 3D representation is used as an explanation for behaviour observed in Fig 3 (page 9 line 244). If this is unreasonable due to experimental/methodological difficulties in working with 3D data, then this should be stated here for clarity.
5. Fig 3 caption: "Here, for each cell/ cell pair, the parameter of the control cell was subtracted from the parameter of the cell under perturbation." What does this mean? Which panel(s) does it refer to?
6. Page 9 line 252: "rather large set of quantifiers" is a strange description
7. Page 10: The first paragraph of "Improving tissue explant viability…." (lines 305-313) is very vague. What are the "numerous internal and external influences" mentioned? Or the "many constraints and parameters" which could be better controlled?
8. Page 12, line 294. "Further visualization tools are needed for this – because "the dark is (almost) never empty"." What is this intended to mean? I'm afraid I don't understand.
9. How many macrophages were considered here? How many were imaged for each stimulus in Fig 3, or for Fig 4?
10. The authors give a detailed description of differences between BMDMs and RTMs at the start of the paper, but subsequent analysis of BMDMs along the lines of Figs 2-4 is missing. Why were BMDMs not studied further? Could this be clarified in the text?
