# Supplementary material for: Cellular morphodynamics as quantifiers for functional states of resident tissue macrophages in vivo
Source: PLoS Comput Biol. 2025 May 29;21(5):e1011859. doi: 10.1371/journal.pcbi.1011859 (PMC12193801; doi:10.1371/journal.pcbi.1011859)
Supplement: Attachment — Submitted filename: ResponseToReviewers.pdf [file pcbi.1011859.s012.pdf]

Daniel Beard and Jason Haugh
Section Editors in Section Cell Biology and Physiology, PLOS Computational Biology

Dear Dr. Beard, dear Dr. Haugh,

Thank you for the opportunity to revise and resubmit our manuscript ‘Cell morphology as a quantifier for functional states of resident tissue macrophages’ to *PLOS Computational Biology*. We would also like to take this opportunity to express our thanks to the reviewers for their feedback and helpful comments for corrections or modifications.

In response to the reviewers feedback, we revised the following main aspects of the manuscript.
1) New experiments – both *in vivo* and on explanted tissue fragments – were performed with improved stimulant concentrations to efficiently induce morphodynamic changes in RTM behavior due to their activation state. The RTMs in those improved experiments could now indeed be successfully analyzed with our pipeline to quantitatively show how chemical stimulation changes the morphodynamics of the cells. And to include a new functionally relevant experiment, we also imaged RTMs from old mice to gain insights into the effects of aging on the morphodynamics of the cells.
2) Furthermore, the list of cell morphology quantifiers used was extended to emphasize that us not being able to clearly distinguish the shape of RTMs in pathological processes was not due to an inadequate choice of quantifiers but due to an innate heterogeneity of the morphodynamics of unperturbed macrophages. To accentuate the advantage of the chosen parameter set – namely being rather intuitive as well as easily human-interpretable – even further, a supplemental text was added describing the chosen quantifiers of cell morphology in more detail.

To better reflect the importance of cell dynamics for this study, we propose changing the title to: "Cellular morphodynamics as quantifiers for functional states of resident tissue macrophages *in vivo*". Furthermore, we added Eric Greto, Anna Möller and Oliver Aust to the list of authors, as they were heavily involved in performing the new experiments and improving the image segmentation pipeline.

This letter contains a detailed response to all of the reviewers comments. We believe the revised manuscript fully addresses the reviewers comments and overall has become a stronger paper.

We look forward to hearing from you regarding our revised manuscript. Thank you for your consideration of the revised manuscript.

Sincerely,

Vasily Zaburdaev and Stefan Uderhardt
Friedrich-Alexander-Universität Erlangen-Nürnberg
Cauerstr. 11
91058 Erlangen, Germany
vasily.zaburdaev@fau.de, stefan.uderhardt@fau.de

Reviewer 1

[Comment 1] The title of the manuscript is somewhat misleading for two reasons:
(A) It implies that cell morphology can be utilized to predict the functional states of macrophages, an assertion that is not thoroughly substantiated within the text (refer also to point 2).
(B) The phrase "functional states" evokes expectations of insights into biologically significant macrophage activities, such as distinctions between inflammatory vs. anti-inflammatory, alternatively activated vs. non-resolving, phagocytic vs. non-phagocytic, and migratory vs. stationary states. However, the manuscript does not adequately address these functional states. A title that more accurately reflects the content of the manuscript, such as "Refined Analytical Parameters for the Morphodynamic Assessment of Resident Tissue Macrophages," would be more appropriate. The authors even demonstrate that treatment with LPS, which induces macrophages into an inflammatory state—a particularly critical functional state—does not result in discernible morphodynamic changes that can be identified by their methods within the analysed timeframe. This observation further underscores the discrepancy between the expectations set by the title and the findings presented.

Response: We appreciate the reviewer's thoughtful comments regarding the title of our manuscript. We have carefully considered the points raised and would like to address them as follows:

(A) We appreciate the reviewer's concern about the clarity of our title. We would like to clarify that our intention is not to predict functional states, but rather to use our pipeline to quantify macrophage behavior. However, we do see our study as an important step towards the development of predictive models - but that was not the scope of this work.

(B) We also thank the reviewer for bringing attention to the expectations set by our use of "functional states" in the title. Especially in light of this comment, we have substantially expanded and improved our study during the revision process. We have:

1. Critically re-evaluated our existing imaging data
2. Conducted additional experiments
3. Included a broader range of stimulations to capture various biologically significant macrophage activities

Specifically, we now include:

- Acute pro-inflammatory stimulation with LPS
- Long-term pro-inflammatory polarization with IFN-gamma
- Acute and chronic pro-homeostatic or pro-resolving stimulation with M-CSF and TGF-beta
- Low-dose endosome blockade using YM201636

These additions allowed us to generate and analyze several prototypical scenarios that macrophages might encounter *in vivo*. Importantly, with this expanded dataset and our refined analysis, we are now able to detect significant morphodynamic differences between these various functional states.

We believe these improvements substantially strengthen our study and better justify the use of "functional states" in the title. The revised manuscript now provides insights into the morphodynamic characteristics of inflammatory, anti-inflammatory, and homeostatic macrophage states, addressing the reviewer's initial concerns.

Finally, we now also propose a new title “Dynamic morphological signatures reveal functional states of resident tissue macrophages in vivo” to make a stronger focus on the dynamic nature of the analyzed features.

[Comment 2.1] Although the authors demonstrate that certain artificial interventions can alter macrophage morphology, there is a lack of rigorous evaluation regarding the predictive capabilities of these morphodynamic changes, contrary to what the title and various assertions within the manuscript suggest. For instance, statements like "We used those features to quantitatively differentiate cells in various functional states," and "... helps distinguish between physiological and pathological cell states," imply a level of predictive accuracy that is not sufficiently substantiated by the data.

Response: Our study establishes a framework for quantifying and distinguishing RTM behavioral motifs at the population level, rather than predicting individual cell behavior. We have critically revised the manuscript to eliminate any language suggesting predictive capabilities. Our goal is to provide a foundation for characterizing RTM morphodynamics, not predicting specific behavioral states.

Regarding RTM behavior predictability in general, we found that naive RTMs display a wide range of behavioral motifs in resting states, which we use to define a physiological morphospace of potential morphodynamical patterns. Notably, upon non-extreme stimulation, RTM populations shift within this morphospace but generally remain within its boundaries. Only in extreme scenarios, such as aging, do we observe shifts outside this defined morphospace. Our data suggests that individual RTMs cannot be reliably distinguished based on morphodynamics alone, but only at the population level, as their individual behaviors fall within the native morphospace.

[Comment 2.2] The functional implications of detecting a "round" macrophage within the tissue are not clearly defined. Does it indicate that the cell is in an environment where the mouse has been overdosed with isoflurane? Does it suggest a deficiency in pinocytosis? Alternatively, it could imply other conditions, such as the cell preparing for division, losing attachment to the extracellular matrix (ECM), or residing in a low-adhesive tissue environment (see work of Maridonneau-Parini, e.g. PMID: 22999511).

Response: In this study and previous work (Uderhardt et al., Cell 2019; Freeman et al., Science 2020), we consistently observed that rounded cell shapes correlate with reduced dynamics, impaired stress responsiveness, and deficiencies in pinocytotic activity. While alternative possibilities exist as suggested here, we never detected RTMs losing attachment to the matrix upon stimulation or becoming migratory (and on a personal note: we tried a lot! Not part of this study). We acknowledge that isoflurane overdose could potentially impact cell morphology, and we decided to remove the isoflurane data as it did not represent a physiological scenario. The work on macrophage adhesion in different tissue environments is

relevant, but our results more closely align with roundness indicating a broader functional deficit, given that the cells are firmly attached initially, and we can't detect acute changes to the ECM within our optical system's resolution capacity. We tried to clarify our interpretation throughout the revised manuscript to address potential confusion.

[Comment 3] A critical aspect that remains unexplored in the study is whether the observed effects of the treatments on macrophage morphology are the result of direct cause-effect relationships or are mediated through indirect influences on the extracellular environment. The question arises whether the impact of YM201636 treatment on macrophages could be secondary to its effects on the tissue environment. Similarly, the observed consequences of isoflurane overdose and tissue hypoxia warrant scrutiny to determine if these responses are directly attributable to macrophages or are indirect outcomes of environmental changes.
In this context, it would have been prudent for the authors to also document and present data on the second harmonic generation (SHG) signal from the connective tissue. This additional analysis would ensure that the extracellular matrix (ECM) environment surrounding the analyzed cells is consistent across samples. Previous research by the Lemmermann lab (PMID: 35343899) has demonstrated that macrophage morphodynamics are significantly influenced by their interactions with the ECM.

Response: We appreciate the comments regarding potential indirect effects on macrophage morphology. After careful consideration, we have made several adjustments to address these concerns.

Firstly, we have decided to remove the isoflurane data from our study (see above). We acknowledge that the observed effects could indeed be multifactorial, and their inclusion might introduce unnecessary complexity to our findings.

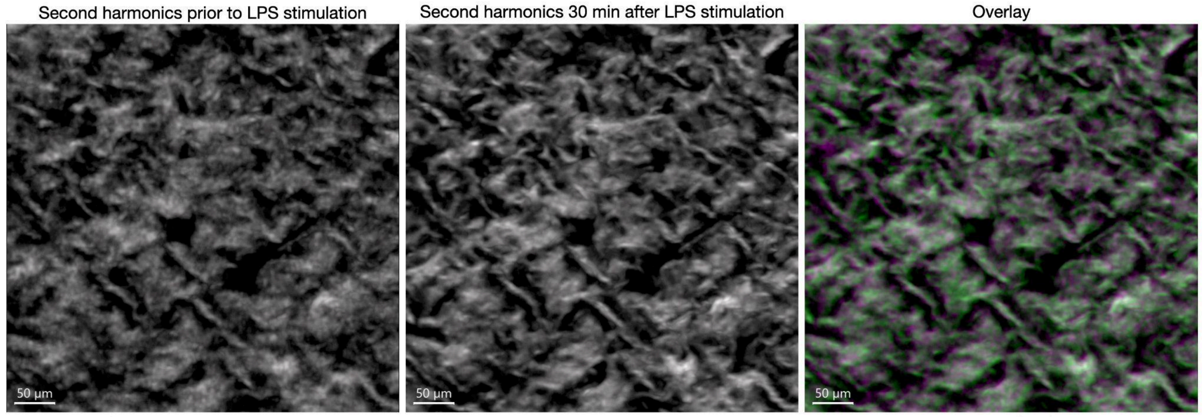

Representative in vivo microscopy images showing 2-photon-generated second harmonics before and 30 minutes after LPS stimulation.

Regarding the possibility of environmental changes influencing macrophage behavior, our current data and previous work do not support this notion. In our earlier studies (Uderhardt et al., Cell 2019; Freeman et al., 2020), we demonstrated that fibroblasts in the peritoneal serosa remain static upon various interventions (including laser damage), at least within the timeframe of our experiments. To further address the reviewer's concerns about potential changes in the extracellular matrix, we have re-examined our existing imaging data, which includes second harmonic generation (SHG) signals. We now provide representative images of the collagen architecture before and after LPS stimulation. While the dynamic nature of intravital imaging makes pixel-perfect overlays challenging, our analysis does not reveal any

substantial changes in or displacement of collagen structure upon stimulation. This finding reinforces our conclusion that the observed morphodynamical changes are primarily intrinsic to the macrophages rather than a result of widespread structural alterations.

It's important to note that the peritoneal serosa, particularly the submesothelial space, exhibits a relatively simple architecture devoid of significant anatomical restrictions or compartmentalization (such as capsules, vessels or ligaments/tendons). This hallmark gives us confidence that all macrophages in our study are situated in at least anatomically similar environments, minimizing the likelihood that their behavioral changes are due to varying physical constraints.

[Comment 4.1] The initial comparison made between BMDMs moving on two-dimensional culture dishes and serosal macrophages within tissue is notably problematic and, to an extent, unfair. In the in vitro setup described, the macrophages are provided with an environment that allows a high degree of freedom, naturally leading to behaviors characteristic of highly migratory cells. Prior research (e.g., PMID: 35343899) has conducted side-by-side comparisons of macrophage morphologies in three-dimensional Matrigel environments versus mouse tissue. These studies have successfully replicated the physiological morphodynamic states of tissue-resident macrophages in three-dimensional setups that offer a lower degree of freedom. As such, Figure 1 is not only misleading but partially incorrect in its conclusions and should be excluded from the manuscript.

Response: We acknowledge that the initial comparison between bone marrow-derived macrophages (BMDMs) and resident tissue macrophages (RTMs) was not optimal. Our intention was to illustrate the stark morphodynamic differences between macrophages *in vitro* versus *in vivo*, underscoring the necessity of our intravital imaging platform for studying macrophage behavior under physiological conditions. In light of the concerns raised by all reviewers, we have decided to replace Figure 1 in the manuscript. Instead, we now focus on highlighting the anatomical composition of the tissue, which provides a more relevant context for understanding RTM behavior in situ. With this, we present a more accurate and nuanced picture of the peritoneal serosa as a model platform for studying RTM biology.

[Comment 4.2] Additionally, the analysis in this paper focuses solely on serosal macrophages, representing just one type of RTM. This specific focus should be clearly stated and maintained throughout the manuscript. It is inappropriate to generalize the findings to all types of RTMs, which can vary significantly in their ontogeny and dynamics and include a mix of macrophages, some of which may be more motile bone marrow-derived macrophages.

Response: This criticism is valid and we have taken steps to address it in our revised manuscript. We have clarified throughout the paper that our study focuses specifically on serosal macrophages as a model system for resident tissue macrophages (RTMs). We recognize that RTMs in different tissues can vary significantly in their origins and behaviors, and have been more precise in our language to avoid overgeneralizing our findings.

We also expanded our discussion to acknowledge the potential impact of macrophage ontogeny on our observations. As a side note, preliminary data, though not included in this study, indicates that by 30 weeks of age, approximately 20% of peritoneal RTMs are bone marrow-derived. This finding may contribute to the marked changes in morphodynamical motifs we observe in aging animals, and we will follow this aspect up in consequent studies.

We have also expanded our discussion to acknowledge that while our approach could potentially be applied to RTMs in other tissues, the peritoneal serosa offers distinct advantages as an experimental platform. Its accessibility and structure allow for high-resolution imaging and versatile experimental manipulations that may not be feasible in other tissue contexts. This addition emphasizes the strengths of our model system while recognizing its limitations in representing all RTM populations.

[Comment 5] The authors introduce adjusted analytical parameters for evaluating morphodynamic characteristics of cells, yet they do not convincingly demonstrate that these new parameters offer any advantages over the traditional parameters used to describe cell morphology, such as roundness, sphericity, ellipticity, branching, protrusion length, sampling activity, etc. The utility and superiority of these novel parameters remain questionable, as the paper lacks a direct comparison to show that these adjustments provide a clearer or more predictive analysis of cell morphology under various conditions.
One might argue that the traditional morphological parameters could also effectively identify significant morphological transformations, such as those induced by extreme conditions like isoflurane overdose or the chemical inhibition of pinocytosis. Without a thorough comparison that highlights the enhanced sensitivity or specificity of the proposed parameters in detecting subtle or complex morphodynamic changes, it remains unclear why the new parameters should be preferred over the established ones.

Response: Our goal was not to introduce novel parameters, but rather to apply established morphological descriptors to a previously unquantified biological scenario - dynamic RTM behavior in vivo. In fact, the parameters we utilized are closely related to traditional measures. For instance, our "circularity" metric is equivalent to the well-established roundness or compactness measures. Similarly, our "aspect ratio" corresponds to ellipticity or eccentricity in other literature. We deliberately focused on automatically-extractable parameters to enable high-throughput analysis and eliminate potential human bias. This initially precluded measures like branching that requires manual assessment. However, we did capture sampling activity by analyzing temporal changes in morphology quantifiers. We also introduced protrusion length as an additional automatically-derived metric relevant to RTM function. While extreme perturbations like isoflurane overdose may be detectable with cruder measures, our quantitative approach enables detection of subtle changes and population-level shifts in RTM morphodynamics. This granular analysis reveals biologically meaningful differences that might be missed by more simplistic categorization.

Reviewer 2

Major comments

[Major comment 1] In my assessment, this study is very interesting and potentially rewarding, but it is quite unclear what really has been achieved, compare e.g. with Ref. 6 on the same system, which had a much clearer outcome. In the end, the main achievement seems to be that a pipeline has been established for quantitative analysis. The results section is a mixture of method description and phenomenological observations but does not provide much mechanistic insight. Let’s take the central statement that “Qualitative morphology changes of RTMs can be deduced from quantitative cell shape measurements”. Here it is shown that some pharmacological interventions lead to measurable changes in the shape features. The backdrop here however seems to be the expectations of the authors of what they know should happen under these conditions. At least this is my understanding of the section title after repeated reading. What is obviously missing here is some new or mechanistic finding that has not been described before.

Response: We appreciate the reviewer's thoughtful assessment of our study. We would like to refer to our response to [Comment 1] of Reviewer 1, which addresses similar concerns.
After careful consideration, we have thoroughly revised and enhanced our manuscript. We have included additional experimental conditions and further refined our analysis pipeline. These improvements have allowed us to make several critical observations that have not been previously described for macrophages *in vivo*. One of our key findings is that the dynamic behavior of resident tissue macrophages (RTMs) is represented in a resting morphospace that exhibits a wide heterogeneity of potential behavioral motifs. This observation provides new insights into the versatility and adaptability of these cells in their native environment and goes beyond the state-of-the-art. Furthermore, we discovered that inflammatory stimulation induces specific changes in these motifs and constrains their distribution towards a more defined and uniform phenotype. Importantly, we observed that these changes occur within the boundaries of the original morphospace, indicating that the cells do not adopt entirely new dynamic behaviors or shapes. This finding suggests a fundamental principle in how RTMs respond to inflammatory signals while maintaining their core functional capabilities. In contrast, we found that only extreme or definitive alterations can indeed change this morphospace, as evidenced by our observations in aged mice. This discovery highlights the potential long-term impacts of aging on RTM function and behavior, which could have significant implications for understanding age-related changes in immune responses and tissue homeostasis.

[Major comment 2] In summary, it is hard to recommend acceptance at the current stage. In my view, this manuscript can be improved considerably in different directions, depending on how the authors want to continue with this project. For example, they could form some mechanistic hypothesis for RTMs and prove this with their pipeline. Why cannot they use the 3D image information to identify periods of medium take-up (which seems to be a major function of these cells, which monitor interstitial fluids) and mechanistically connect this to other changes in the morphodynamics?

Response: We thank this reviewer for the suggestions and constructive feedback. While we acknowledge that truly mechanistic studies using intravital imaging present significant challenges, we are actively developing a novel explant imaging platform that offers greater experimental versatility to address such questions in future work. Instead, we have substantially enriched our current study by refining our analysis process and incorporating a range of *in vivo* challenges and stimulations that more closely resemble potential real-life scenarios for RTMs. The stimulants used now include anti-inflammatory agents (M-CSF and TGF-beta), pro-inflammatory agents (LPS and IFN-gamma) and an endosome-blocking agent (YM201636).
Our revised manuscript now presents a unique interventional *in vivo* imaging approach and a rich dataset of RTM morphodynamical motifs under various conditions. This comprehensive analysis lays a solid foundation for further theoretical studies, including the development of predictive modeling approaches.

[Major comment 3] Alternatively, they could develop this project further as a methods paper, but then they should go beyond the state-of-the-art with standard software. I do not agree that ML and DL based methods are "emerging" like written in the discussion, they are already standard. I recommend to look into DL methods and to train a deep network on a fraction of the data and then to demonstrate that for the other fraction a functional state can be predicted from the shape features as suggested by the title. Maybe the authors have different ideas how to proceed in the methodological direction, but at the current state of affairs, they seem not to unleash the full potential of the available methods in this field.

Response: We agree that machine learning and deep learning methods have become standard tools in bioimage analysis, and we have removed the term "emerging" from our discussion to reflect this. We also concur that AI-driven segmentation approaches could potentially enhance analysis quality and throughput for studies like ours. However, it is important to note that during the course of this study, we extensively evaluated various ML/DL applications, including training on our own dataset. Despite these efforts, we found that existing methods struggled to reliably detect and segment the complex, highly dynamic morphologies of macrophages *in vivo*. For instance, even state-of-the-art tools like Cellpose3 tended to generate overly simplified, blob-like masks that failed to capture the intricate protrusive phenotypes we observed. Other approaches like PlantSeg or SAM2 often resulted in oversegmentation, necessitating laborious manual corrections. While AI-based segmentation remains a promising avenue, the unique challenges posed by our experimental system required us to develop a custom pipeline to accurately quantify macrophage morphodynamics. That said, we recognize the value in further advancing segmentation methods for complex cellular shapes. To that end, we have now compiled a large dataset of annotated 2D+time macrophage masks from our experiments. In follow-up studies, we are actively pursuing collaborations with AI image analysis experts to leverage this data and develop improved segmentation algorithms tailored for highly dynamic cells like RTMs. Our modular pipelines allows us to easily incorporate new segmentation results. We address this issue now in the discussion section of the revised manuscript.

Minor comments

[Minor comment 1.1] The results section is full of literature survey and explanations that are not really results, but should go into e.g. the introduction. The authors should ask themselves what really is a result and what is existing knowledge.
This is especially true for the explanation on shape analysis from pages 4-6, which definitely are not results. Moreover, this list is not very systematic and it is not explained why e.g. Fourier descriptors are not used.

Response: We agree that some explanatory material could be better placed in other sections of the manuscript to improve clarity and flow.

The introduction of shape quantifiers has been moved to the introduction section, as suggested. This provides readers with the necessary background earlier in the paper. However, we have retained the mathematical definitions of specific morphology quantifiers in the results section, as these are directly referenced and discussed in subsequent analyses. We believe this placement allows readers to easily connect the definitions to their applications. Regarding the brief literature review on chemical perturbations and their effects on macrophages, we have kept this in the results section. Our intent was to provide context for non-expert readers when introducing the effects of these chemicals on cell shape. We believe this placement enhances the accessibility and interpretation of our findings.

We acknowledge that our list of quantifiers is not exhaustive. We have clarified our approach in the revised manuscript, explaining that we adopted the classification of parameters from Mingqiang et al., focusing on those describing overall cell shape. Our selection criteria emphasized human-interpretability and ease of automatic extraction. We have expanded on this rationale and provided a more detailed interpretation of the quantifiers as supplemental material. Concerning specific shape analysis methods like Fourier descriptors, we have added a brief explanation of why certain techniques were not used, considering factors such as applicability to our specific cell types and compatibility with our analysis pipeline.

[Minor comment 1.2] As an example where such a shape analysis led to mechanistic insight, I point to Keren, Kinneret, et al. "Mechanism of shape determination in motile cells." Nature 453.7194 (2008): 475-480. Can something similar be done here for the RTMs?

Response: We appreciate the reviewer's suggestion to consider the approach used by Keren et al. in their study of shape determination in motile cells. While we agree this work provides an excellent example of how quantitative shape analysis can lead to mechanistic insights, there are important differences in our experimental system that preclude directly applying their methods. Keren et al. were able to visualize actin filaments at the leading edge of keratinocytes, which was crucial for connecting shape changes to underlying cytoskeletal dynamics. In contrast, our study focuses on resident tissue macrophages (RTMs) imaged *in vivo* in the peritoneum. Currently, technical limitations (e.g., optical resolution, available probes or genetic models) prevent us from directly correlating our shape quantifiers with real-time cytoskeletal rearrangements. However, we hope that the development of more versatile explant imaging platforms will help to overcome this limitation. So while we cannot achieve the same level of mechanistic insight as Keren et al. at this time, our shape analysis of RTMs still provides valuable information about their morphodynamics in different functional states.

[Minor comment 2] Fig. 1: a quantitative analysis of the motility data is missing, e.g. plots of position or MSD over time.

Response: Figure 1 was replaced entirely due to multiple concerns.

[Minor comment 3] The sentence on line 179 is grammatically not complete.

Response: Thank you, the sentence was corrected in the revised manuscript.

[Minor comment 4] Language is very strong and should be tuned down, e.g. “These analyses provided unprecedented insight into the dynamic behavior of RTMs”. This sentence is hard to justify given that the shape analysis did not give much insight due to strong variability.

Response: We revised the manuscript, adjusted the language, added to new analysis steps and data, and can now provide novel biological insights into *in vivo* RTM behavior upon various conditions.

[Minor comment 5] Discussion: to what other systems than RTMs could this pipeline be applied in the future?

Response: The main features of a perfect cell type candidate to be studied with this pipeline would be:

- The cell would have to be stationary, so that a fixed cell area can be detected as well as to facilitate segmentation and cell tracking.
- Dynamic activity based on formation of temporary protrusions.
- A possibility for either *in vivo* imaging of the cells within their niche resp. surrounding tissue and/ or excellent explantation methods to capture both the morphology and dynamics of the cells as close to their physiological state as possible.
- Highly individual cell morphologies (unlike cell shapes in densely packed tissues)

There are not many examples of cells that would fall into this category. E.g., dendritic cells or neutrophils are migratory, neurons on the other hand do not display sampling-like behavior. Even if this pipeline were limited to studying tissue macrophages, its importance would remain significant. Macrophages constitute the most abundant immune cell population in native tissues (see Sender et al.. 2023. The total mass, number, and distribution of immune cells in the human body. *Proc Natl Acad Sci U S A* 120:e2308511120. doi:10.1073/pnas.2308511120), yet our understanding of their diverse functions across various microenvironments and tissue compartments in health or disease remains incomplete. Dynamic analyses can bridge the gap between transcriptional phenotyping and actual *in vivo* function, providing crucial insights into macrophage biology.

Reviewer 3

Main comments

[Main comment 1] How correlated are the summary statistics chosen for this data? E.g. the curves in Fig 2D look highly correlated, how much value is added by measuring all these metrics?

Response: The features plotted in Fig 2D are indeed rather correlated for this cell, which is also measurable by the correlation coefficient (see Table R1):

Table R1: Pearson's correlation coefficients for the cell morphology quantifiers depicted in Fig 2 of the initially submitted manuscript.

Cell in Fig 2	Solidity	Convexity	Circularity	Aspect ratio
Solidity	1			
Convexity	0.55	1		
Circularity	0.79	0.83	1	
Aspect ratio	-0.49	-0.79	-0.49	1

However, this strong correlation cannot be seen for every cell, see for example another cell from the control condition in figure Fig R1, also with calculated correlation coefficients in Table R2.

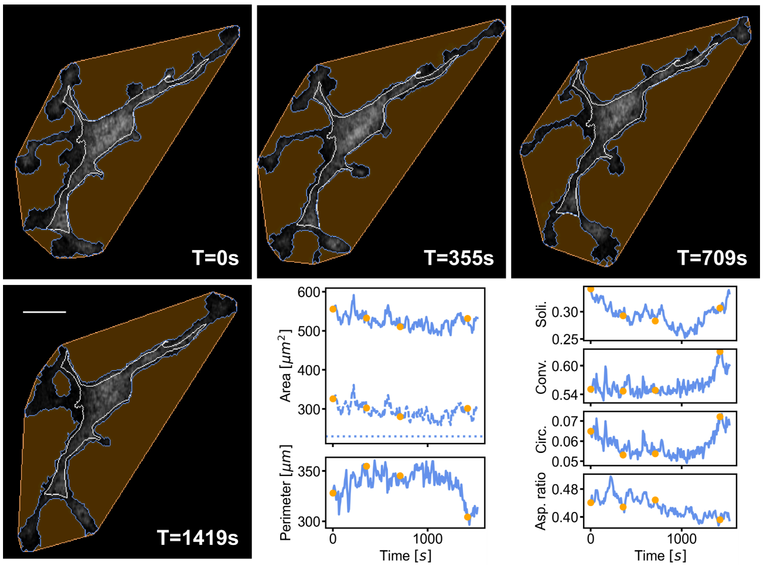

Fig R1: Figure analogous to Fig 2 of the initially submitted manuscript, but for another cell of the control condition.

Table R2: Pearson's correlation coefficients for the cell morphology quantifiers depicted in Fig R1 above.

	Solidity	Convexity	Circularity	Aspect ratio
Solidity	1			
Convexity	0.25	1		
Circularity	0.75	0.79	1	
Aspect ratio	0.31	-0.38	0.01	1

For this example, we can see that only the circularity is still correlated to solidity and convexity, which makes sense as the cell area and the cell perimeter are used in the definitions of the parameters.
After reconciling with Figs S3 and S4, every significant shape change seen in the circularity is also seen in the solidity, so it might indeed be possible to actually remove the circularity shape feature completely without losing information when qualitatively assessing macrophage morphology. However when checking the components of the PCA, the mean respectively standard deviation of the circularity is one of the main constituents of PC1 respectively PC2, confirming its relevance even though it is correlated to other parameters.

[Main comment 2] Why were these 4 shape metrics chosen, and not other possible choices? For instance, several others are mentioned in the text, and there are a huge number of shape quantifiers available in the wider mathematical literature that are not discussed (e.g., fractal dimension as a measure of boundary complexity). Given that the metrics chosen here don't seem to distinguish well between different macrophage subtypes (see PCAs in Fig 3 and Fig 4 and comments 6/7 below), it seems strange not to include a wider range of quantifiers.

Response: The set of parameters were chosen for three main reasons. 1) They are already (partially) established in the community to analyze cell shapes and specifically macrophage morphology. However, in previous works macrophage morphology was usually analyzed either *in vivo* but with a fixed sample, or dynamically *in vitro*. Both experimental setups do not allow to quantify RTM behavior in a living organism. Furthermore, 2) the chosen features can be quantified automatically, thus eradicating the human factor as a potential source of error or bias. Therefore some parameters used to characterize RTM morphology such as their branching - which are typically extracted by hand using e.g. ImageJ - were not considered here. Moreover, 3) we were looking for parameters which can be interpreted qualitatively, such that one can have an intuition about how a cell is shaped given its quantification. This is also depicted in Fig. 2, where the qualitative shape changes for different values of the quantifiers are represented.
As we argue in Section “Dimensionality reduction methods for morphology quantifiers show large heterogeneity of unperturbed RTM shape and dynamics” (called “Dimensionality reduction reveals extensive RTM morphospace and stimulus-specific constraints” in the revised manuscript), the quantifiers do distinguish cells activated by inflammation-inducing stimuli. As to why the quantifiers do not distinguish cells which were perturbed from cells in a

resting state, we speculate that there is a physiological reason why control/resting cells show a large heterogeneity in their morphology. However, to confirm this large heterogeneity we considered more morphology features – namely the protrusiveness, the surface angularity, the length of the maximal cell protrusion as well as the dynamic area change – which also show a large spread of the control cells thus confirming our previous findings. The PCA of the different conditions has not significantly changed to allow a better differentiation between different cell states from the control condition which strengthens our hypothesis that a large morphodynamic heterogeneity of RTMs has a morphological reason and is not only visible due to our originally more limited parameter set.

[Main comment 3] The move from time series of statistics to aggregate statistics is not well clarified here – for instance, in Fig 3 the mean of the perimeter is used, but the Std of solidity and circularity. Why were these metrics chosen? How well do they capture the variation in the time series? Why are linear trends of the time series taken when they are seen in Fig 2 to be cyclical?

Response: The boxplots in Fig 3 are chosen as an exemplary subset of all the shape features plotted in Figures S2, S3 and S4, showing (at least) one example of each summary statistic (mean, std, trend). We tried to clarify that with an addition in the main text of the revised manuscript. As seen in Figure R1 answering the first comment, not all (actually only very few) RTMs show this clear cyclical behavior in their shape features, thus a linear trend seems appropriate to describe the data sets.

[Main comment 4] Figure 3 is used to support a wide range of claims on pages 6-9, but much relevant information is instead in the SI (Fig S2) and it is not clear that the interpretations given here are the only way of interpreting these statistics. Clarifying the interpretations of the statistics used in this paper would be extremely helpful to demonstrating that these boxplots (most of which show no statistical significance) support the authors' interpretations regarding macrophage morphology. Otherwise we are left to rely on statements like "it was visually apparent that x" which are then supported with statistics that may support this interpretation, or may support some other interpretation. See, for example, lines 238-241. Increased roundness is a possible interpretation of the changing perimeter etc mentioned here, but there is no discussion in the text as to whether another change in morphology could also give rise to these changes in statistics.

See also page 7: "We have shown that our set of parameters were indeed capable of differentiating between RTMs perturbed by different chemical stimuli - some of the features do so on the population-level while others are more useful in direct comparison at the cell-level" – I don't understand how Fig 3 supports this?

Response: As mentioned in response to [Main comment 3], Fig 3 is meant to show an exemplary subset of quantifiers as showing all plots supporting our analysis of the cell shape in the main text seemed not feasible. Thus, this statement is indeed supported by Fig 3, but most importantly in combination with Fig S2.
In the added supplemental material, a detailed description of the chosen morphology parameters is given, with possible interpretations of changes in the parameter values.

[Main comment 5] S2 Fig – S4 Fig – some captions would be extremely helpful in clarifying what these images show.

Response: The captions of those supplementary figures (now S2, S4 and S6) were extended to include more information on the cell populations compared - the definition and interpretation of the shown parameters is given in an additional supplemental text.

[Main comment 6] Fig 3 panels I and J – how much of the variance do PC1 and PC2 explain? Would using 3 principal components lead to better separation between the classes? Does clustering in the high dimensional space (rather than the reduced dimensional projection) allow the clusters to separate?

Response: Using 3 or more principal components for comparing all five *in vivo* populations (Control, LPS, M-CSF, Iso and YM) does not lead to a better separation of the classes (see e.g. the 3-dimensional PCA attached in Fig R2 here). As we argue in section “Dimensionality reduction reveals extensive RTM morphospace and stimulus-specific constraints” in the revised manuscript, we speculate that chemical perturbations actually narrow down the large variety of cell shapes to a subset, which possibly is also visible in the Ctrl population, meaning that there might be cells in the Ctrl populations which are already partially adapted to answer to specific alarm signals in the tissue. For the new data with adapted cell morphodynamic parameters, we checked again if a three dimensional PCA would result in a better separation of the different cell populations, which was not the case (see Fig R2).

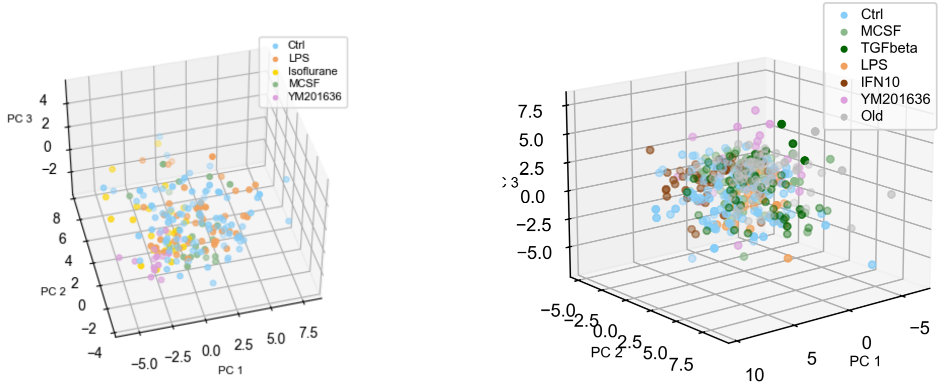

Fig R2: LEFT: Three dimensional PCA of the data shown in the 2D PCA in the original manuscript in Fig. 3. RIGHT: Three dimensional PCA of the data shown in the 2D PCA in the revised manuscript in Fig. 3

[Main comment 7] The PCA plots shown in Fig 3I and Fig 4F both seem to show no substantial difference between the classes. It seems likely to me that this is because the features chosen to define the high dimensional space do not adequately describe shape differences present between the macrophages compared (see comment 2 above). The lack of significant differences in Fig 3 B-G seems to support this. How important are the features the authors have selected here? Why were these features chosen to summarise the time series in Fig 2 described and not others?

Response: It is indeed possible that the PCA does not show substantial differences between the classes because of an inadequate choice of features. To test this hypothesis, more features were chosen to describe the morphodynamics of macrophages – namely (surface) angularity, protrusiveness, the length of the longest protrusion of the cell as well as

the dynamic area change – to test if other quantifiers may measure significantly different morphodynamics for RTMs in different states. However, adding those quantifiers to the PCA still did not lead to a good separation of the classes, especially if one would try to cluster the unperturbed control population from activated cells. Therefore, we see our hypothesis supported that resting RTMs may show a large morphodynamic variability, which is narrowed when they are being perturbed.

[Main comment 8] The authors describe their features repeatedly as “human-interpretable”. It would be useful to have some comment on how these features can be interpreted, particularly when interpreting combinations of shape quantifiers. What information does each quantifier give? When do they give similar or different information, and how does this affect the interpretation? (See also comment 4 around the interpretation of Fig 3.)

Response: As mentioned in the response to comment 4, a more detailed analysis of the single parameters is provided as supplemental material, thus hopefully clearing their interpretation up.

Minor comments

[Minor comment 1] Fig 2C: Although explained in more detail in the main text, I found the different area curves confusing here. Could they be colour coded to relate them to the relevant outlined areas in Fig 2A? If the fixed area is constant, what is gained by plotting both the total area and mobile area – is the total area just the sum of the other two curves, one of which is constant?

Response: By color-coding the single area curves and thus adding more colors to the plots and snapshots, there is the risk of making the overall figure even more confusing. While it is true that nothing is gained by plotting the mobile area (as it has the same shape as the whole area, and mobile and fixed area indeed add up to the total area), it might have been more confusing if this parameter later shows up in the supplementary figures (S2, S4), but was not plotted in Fig. 2. Hopefully the adjusted figure caption for Fig. 2 clarifies what the single curves mean.

[Minor comment 2] Page 2: “Although cell shape analyses have been used to characterize macrophages under various conditions, most of the approaches used were either not designed to assess the dynamics behavior of peripheral RTMs over time or did not take into account the inhere characteristics of sessile RTMs in vivo.” This sentence seems to be a brief discussion of previous literature, which is not cited here. Which previous approaches are the authors referring to? Can citations to them be added here?

Response: Examples of previous literature quantifying the shape of RTMs were added here (or rather moved from another part of the manuscript.)

[Minor comment 3] Fig 1 provides a clear demonstration that in these data the macrophages observed in vivo and in vitro display different motile vs sessile behavior. Is this representative of all such in vivo vs in vitro phenotypes? Have any RTMs in vivo been observed to be motile, and do RTMs placed in vitro display a motile or sessile phenotype? Some minor expansion on the limit of these experiments (or supporting references here or in the introduction if this behavior is well-known in the literature) would be helpful to clarify.

Response: There exist indeed more motile RTMs *in vivo*, either due to chemical stimulation, or because the RTMs reside in different tissue niches. However, the comparison of BMDMs to RTMs was mainly meant as an introduction to the different possible experimental set-ups, which lead to very different morphodynamics of the cells. However, as this was not clear from Figure 1, it was replaced to instead show the tissue niche of peritoneal RTMs - the make-up of the surrounding tissue has a major impact on how the cells can perform their normal functionality and thus shows physiological limitations of the sampling dynamics of RTMs.

[Minor comment 4] The analysis is conducted in 2D, but 3D data is available, with a brief justification given for working with maximum intensity projections (page 4). It would be useful if possible to see a brief quantitative justification for this (e.g., supplementary images showing the symmetry in the z-direction, or quantification showing that including morphology in the z-direction does not add predictive value), especially given that lack of 3D representation is used as an explanation for behaviour observed in Fig 3 (page 9 line 244). If this is unreasonable due to experimental/methodological difficulties in working with 3D data, then this should be stated here for clarity.

Response: Hopefully the revised figure 1 of the manuscript which includes a cross-section of the peritoneum, helps to show that the geometry of the tissue does not allow the RTMs to change their shape in the z direction significantly, which is why using 2D data for analysis should be justified. Moreover, original 3D data was in fact used by us for advanced image segmentation pipeline as now detailed in the Materials and Methods section.

[Minor comment 5] Fig 3 caption: “Here, for each cell/ cell pair, the parameter of the control cell was subtracted from the parameter of the cell under perturbation.” What does this mean? Which panel(s) does it refer to?

Response: This sentence referred to panels (F, G) in the initial manuscript. It refers to how the identical cells - first unstimulated, then chemically stimulated - were analyzed. However, as this type of experiment was not repeated for the newly added data, this type of analysis was also removed from the revised manuscript.

[Minor comment 6] Page 9 line 252: “rather large set of quantifiers” is a strange description

Response: This expression was changed to include the actual number of quantifiers.

[Minor comment 7] Page 10: The first paragraph of “Improving tissue explant viability....” (lines 305-313) is very vague. What are the “numerous internal and external influences” mentioned? Or the “many constraints and parameters” which could be better controlled?

Response: We adjusted the manuscript and better detailed the problems that can arise with intravital imaging experiments.

[Minor comment 8] Page 12, line 294. “Further visualization tools are needed for this – because “the dark is (almost) never empty”.” What is this intended to mean? I’m afraid I don’t understand.

Response: Here we mean the surrounding tissue, which is not labeled and thus displayed in the given video (and also in the snapshots) as just black (dark) background, when in reality there are other cells, ECM, etc. that influence the shape and movement and dynamics of the RTMs. This is also visible in the revised Figure 1, where a cross-section of the peritoneum is displayed.

[Minor comment 9] How many macrophages were considered here? How many were imaged for each stimulus in Fig 3, or for Fig 4?

Response: While the sample sizes for the different conditions are also given in Table 1 (page 16 of the manuscript), this information was also now added to the main text and the figure captions.

[Minor comment 10] The authors give a detailed description of differences between BMDMs and RTMs at the start of the paper, but subsequent analysis of BMDMs along the lines of Figs 2-4 is missing. Why were BMDMs not studied further? Could this be clarified in the text?

Response: As already mentioned in response to minor comment 3, the intention of Figure 1 was to show the stark influence that experimental set-ups *ex vivo* have on the size, shape and motility on RTMs, thus arguing why it is so crucial to analyze morphodynamic features of RTMs *in vivo* to gain insights into physiological relevant processes. In the new manuscript, figure 1 was changed so that the comparison of RTMs and BMDMs is no longer included.
