# Supplementary material for: Cellular morphodynamics as quantifiers for functional states of resident tissue macrophages in vivo
Source: PLoS Comput Biol. 2025 May 29;21(5):e1011859. doi: 10.1371/journal.pcbi.1011859 (PMC12193801; doi:10.1371/journal.pcbi.1011859)
Supplement: Attachment — Submitted filename: CoverLetterResubmission2.pdf [file pcbi.1011859.s013.pdf]

Philip K Maini and Jason Haugh
Academic and Section Editors in Section Cell Biology and Physiology, PLOS Computational Biology

Dear Dr. Maini, dear Dr. Haugh,

Thank you again for the opportunity to revise and resubmit our manuscript 'Cellular morphodynamics as quantifiers for functional states of resident tissue macrophages *in vivo*' to *PLOS Computational Biology*. Furthermore, we thank the reviewers again for their feedback and detailed comments for corrections or modifications.

In response to the reviewers feedback, we revised the following main aspects of the manuscript by including new experimental data (1) as well as quantification of our observed behavioral patterns (2).

Ad 1.: Regarding Reviewer #1's request for additional data to demonstrate the functional differences between our differently stimulated macrophages, we acknowledge the value of the suggested uptake assays and comprehensive transcriptional response analyses. However, we respectfully note that such extensive experiments would go beyond the scope of the current manuscript, both in terms of experimental effort and associated costs since we are still dealing with complex *in vivo* environments. Nevertheless, we have addressed this important concern by including new data that demonstrate distinct molecular responses of macrophages to our short-time stimulation protocols at the level of transcription factor activation. For this, we performed immunostaining of PFA-fixed tissues for five key transcription factors (STAT1, STAT3, Akt, Foxo3a, p65) in macrophages assessing subcellular localization upon our different stimulation settings. For instance, we now clearly show that specifically upon LPS stimulation, macrophages display a prominent accumulation of the NFκB transcription factor subunit p65 in the nucleus, which signifies activation and initiation of downstream transcriptional responses. These additional data not only provide a comprehensive collection of molecular signatures of macrophage responses in vivo but also establish the foundation for potentially linking these molecular responses to the dynamic behavioral patterns we observe. We believe these additions substantially strengthen our manuscript while maintaining its focused scope. The new data effectively address the core of Reviewer #1's concern by providing clear evidence of functional differentiation between our stimulation conditions, albeit through a different experimental approach than initially suggested.

Ad 2.: Quantification of the multi-dimensional morphospace of RTMs: we implemented a test to quantitatively assess whether multivariate populations come from the same distribution to determine how much the differently activated RTM populations differ from each other. Here, we managed to quantitatively differentiate RTM populations exposed to different stimuli.

This letter contains a detailed response to all of the reviewers comments. We believe the revised manuscript fully addresses the reviewers comments and overall has become a stronger paper.

Sincerely,

Vasily Zaburdaev and Stefan Uderhardt
Friedrich-Alexander-Universität Erlangen-Nürnberg
Kussmaulallee 2
91054 Erlangen, Germany
vasily.zaburdaev@fau.de, stefan.uderhardt@fau.de

Reviewer 1

In the revised manuscript, the authors present additional datasets in which they globally treated serosal tissues with various stimuli (e.g., pro-inflammatory stimuli like LPS or IFN-gamma, and anti-inflammatory stimuli such as M-CSF or TGF-beta) before using their analysis pipeline to examine the morphodynamics of resident tissue macrophage (RTM) populations.
With these new datasets, the study further suggests that artificially induced polarization of macrophages into “functional” states correlates with distinct morphodynamic phenotypes at the population level.

[Comment 1] However, the study still does not demonstrate that the analysis pipeline can detect, quantify, or identify actual “functional” states of tissue macrophages within a heterogeneous cell population. For instance, it does not allow for the identification of individual inflammatory or anti-inflammatory macrophages in a truly physiological context, such as during a local tissue infection where diverse cell phenotypes would be expected. In their response letter, the authors clarify that their aim was not to predict functional states but rather to quantify macrophage behavior. While useful, this approach falls short of addressing the potential to detect functional states within mixed populations, which would have been a more novel contribution to tissue macrophage biology.

Response: This is correct - we argue that it is not possible to definitively differentiate individual resident tissue macrophages (RTMs) into a pro- or anti-inflammatory state, as these phenotypes and morphodynamic signatures already exist within the heterogeneous control steady-state population. While the goal the reviewer describes—distinguishing RTMs in an inflammatory state within such a heterogeneous population—is indeed highly desirable, our manuscript demonstrates that this is unfortunately not achievable at the single-cell level. Nevertheless, we have successfully shown that cell populations can be distinguished, which we highlight in this second round of revision. Specifically, we have employed the Rosenbaum test to differentiate RTM populations in activated states from both the control state and other activated states, thereby demonstrating that our analysis pipeline can quantitatively detect shifts in ‘functional states’ on a population basis. This population-level approach provides a robust method for characterizing macrophage responses while acknowledging the inherent heterogeneity that precludes reliable single-cell classification.

[Comment 2] Consequently, the manuscript continues to function more as a methods paper, with modest biological novelty. The work feels more like a foundation for training mathematical learning models based on the current datasets. Although the authors now argue for the value of detecting heterogeneity in macrophage populations within untreated, steady-state tissues, I would contend that this finding is largely intuitive and expected.

Response: We respectfully disagree with the assertion that our finding is intuitive or expected. In fact, we initiated this study with the explicit hypothesis that we would observe distinct population shifts upon stimulation. As discussed in the manuscript, the discovery that macrophages within an anatomically homogenous tissue compartment—despite being from a naive, seemingly uniform population—display such a diverse spectrum of morphodynamic traits—including traits observed upon inflammatory or anti-inflammatory stimulation—is novel and unexpected. Such vast heterogeneity likely carries biological significance, as it enables flexible adaptation to various non-homeostatic challenges. Our results reveal that physiological stimulation does not generate entirely new morphodynamic motifs but rather narrows the range of existing motifs into discrete niches within the naive morphospace. We speculate that morphodynamic signatures falling outside this naive

morphospace may indicate non-physiological dystrophic conditions or aging processes. To strengthen our conclusions, we have implemented a new quantification approach in this revision that successfully distinguishes between cell populations. Specifically, we employed the Rosenbaum test to differentiate RTM (resident tissue macrophage) populations in various activated states from both the control state and other activated states. This analysis demonstrates that our pipeline can quantitatively detect changes in 'functional states' at the population level.

[Comment 3] Additionally, the authors have now included data on macrophage populations in aged tissues, showing that these macrophages exhibit different morphodynamic characteristics compared to those in younger tissues. However, the functional implications of these differences remain unexplored.

Response: As described above, our study demonstrates that motifs depart from the established morphospace under non-physiological conditions such as aging, and importantly, that this departure appears potentially reversible. While these findings suggest intriguing possibilities for future research, they represent the extent of our conclusions at this stage of the study.

[Comment 4] It is also notable that several paragraphs still imply that the analytical pipeline can detect, quantify, or identify functional states of macrophages within tissue, despite the study not providing evidence of this capability. This starts with the changed title "Cellular morphodynamics as quantifiers for functional states of resident tissue macrophages in vivo" or the suggested title "Dynamic morphological signatures reveal functional states of resident tissue macrophages in vivo" in response to my previous comments (see response letter). It continues with statements such as "These features allowed for the quantitative and qualitative differentiation of cells in various functional states, including pro- and anti-inflammatory activation and endosomal dysfunction.", "distinction between physiological and pathological cell states and the assessment of functional tissue age" and "distinguish cells in different physiological and pathological states"

Response: We argue that we are able to differentiate RTMs on a population level, as seen in the multiple box plots differentiating between e.g. steady-state RTMs and RTMs in an inflamed state (due to e.g. LPS). Furthermore, we have now included a statistical test which can distinguish cell populations in different functional states (see also our response to Comment 1). However, as we demonstrated with the heterogeneity in the PCA plots, it appears to be impossible to differentiate RTMs on a single-cell level. In the second revision of the manuscript, we clarified that we can (now also quantitatively) distinguish (not predict) RTMs in different functional states. The statements mentioned in the comments were edited accordingly to emphasize the differentiation on the population level

[Comment 5] For a meaningful biological advance, it would be essential for the authors to link specific morphodynamic states to concrete indicators of macrophage functionality, such as transcriptomic profiles, NF-kappaB activation, IL1-beta production, or increased phagocytic activity.

Response: We fully agree and that is something we are currently exploring in different more biology-focused projects. Transcriptomics analysis would fail to detect meaningful differences due to our mostly short-time stimulation, cytokine production would best have to be measured at mRNA levels and bringing phagocytic prey into interstitial compartments for phagocytosis assays as well as finding practical experimental conditions has proven challenging. However, to address this critic we set up experiments with PFA-fixed tissue after stimulation to assess the subcellular localization of key transcription factors such as STAT1, STAT3, NFkB p65, Foxo3a and Akt in response to our five experimental conditions (LPS, IFN-ɣ, TGF-β, M-CSF, YM201636). Specifically, we qualitatively

visualized nuclear accumulation of transcription factors that would indicate their cytoplasmatic activation and subsequent translocation as initiators of transcription. With this we can clearly demonstrate differences at the molecular response level between the different groups as would be expected with subsequent transcriptional polarization into pro- or anti-inflammatory phenotypes, for instance. So we can clearly show a prominent NFkB p65 accumulation in macrophages upon LPS that other stimulations are lacking. These additional experiments provide direct evidence of differential signaling pathway activation in response to our various stimulation conditions that have been acknowledged with downstream transcriptional profiles before. We believe these changes address the concerns while maintaining the focus of our current study on the methodological approach.

[Comment 6] I cannot judge whether the analysis pipeline itself in combination with the descriptive and correlative nature of the current manuscript is sufficient for the scope of Plos Comp Biol. For me, the here provided presentation of novel parameters for morphodynamic cell analysis combined with the analysis pipeline falls into the category of a methods paper.

Response: We strongly believe that we are exactly within the scope of the journal: we used computational methods (image analysis, automatic parameter measurements from those images via the computer, statistical testing of significant changes in cell morphodynamics) to gain new insights into the biology of macrophages (i.e. showing quantitatively the heterogeneity of the unperturbed RTM population, showing the effects of aging, showing the importance of unbiased morphodynamic features and measurements when judging the quality of a tissue explant). We are confident that those results are actually new and of interest to this exact community.

Reviewer 2

[Comment 1] The authors have performed a very substantial revision, as can be seen from the many changes in the markup copy and from the changes in the author list (shift from Zaburdaev to Uderhardt as last author, inclusion of three new authors from the Uderhardt lab). In detail, they have included new experiments, improved the image processing pipeline, removed problematic Figure 1 and the isoflurane data, sharpened their conclusions and clarified the many issues raised by the reviewers. There are now clear biological results and the image processing is put much better into context. In my assessment, the effort was worth it and this manuscript is now very strong. I recommend publication as is.

Response: We thank the reviewer for his kind feedback and are happy that we could address all his previously raised concerns.

Reviewer 3

[Comment 1] Thanks for the substantial changes to the manuscript, which deal with most of my previous comments and make things clearer. I'm still not convinced by Fig 4 and the discussion around it that justifies the lack of clear separation in the PCA plots (if these metrics are good enough to distinguish macrophages based on activation state then it should be possible to see this). However, the argument that in control conditions macrophages display a broader array of morphologies which are then restricted to a region of morphology space on activation is sufficient justification for me to look past it here.

Response: Thank you for your positive feedback. For this revision we decided to add a quantification of the given PCA, or rather the 31-dimensional data space the PCA was based on, namely the Rosenbaum test. With this test, which uses the introduced morphodynamic metrics as a basis, we could quantitatively distinguish RTM populations in different activation states, even though there was no clear separation of populations visible in the PCA.
